# The influence pathways of physical activity on anxiety in university students: a systematic review and meta-analytic structural equation modeling study based on psychological resilience and social support

**DOI:** 10.1186/s40359-025-03915-2

**Published:** 2026-01-12

**Authors:** Chen Ge, Xilin Li, Jiangxuan Li, Chengbo Yang

**Affiliations:** School of Athletic Training, Chengdu Sport University, 1942 North Huhu Road, Eastern New District, Chengdu, Sichuan Province 641418 China

**Keywords:** College students, Physical activity, Psychological resilience, Social support, Anxiety

## Abstract

**Background:**

Previous studies indicate that physical activity significantly alleviates anxiety among college students, and that psychological resilience and social support may mediate this relationship; however, existing evidence remains limited. This study aims to examine the mediating roles of psychological resilience and social support in the relationship between physical activity and anxiety in college students, and to analyze potential moderating factors.

**Methods:**

Eight databases were searched without restrictions on language or publication time to obtain relevant coefficients. Meta-analytic structural equation modeling (MASEM) was applied to verify the mediating roles of psychological resilience and social support. In addition, one-stage MASEM was employed to assess the moderating effects of female proportion and sampling methods.

**Results:**

Twenty-nine articles were included, comprising a total sample of 36,159 participants and 90 effect sizes. Physical activity had a significant negative effect on college students’ anxiety (β = –0.262, 95% CI: –0.281, –0.243). Psychological resilience (β = –0.152, 95% CI: –0.163, –0.142) and social support (β = –0.048, 95% CI: –0.055, –0.042) served as mediators. Female proportion and sampling methods exhibited moderating effects on the model. Sensitivity analysis and publication bias tests supported the robustness of the findings.

**Conclusion:**

Increasing college students’ physical activity levels effectively reduces anxiety, and physical activity indirectly alleviates anxiety through enhanced psychological resilience and social support. Future research should focus on causal relationships among variables and on differential effects of gender and sampling methods, thereby providing more effective mental health support for college students.

**Trial registration:**

This study has been registered with the International Prospective Register of Systematic Reviews (PROSPERO) (registration number: CRD420251129622).

**Supplementary Information:**

The online version contains supplementary material available at 10.1186/s40359-025-03915-2.

## Introduction

Anxiety (ANX) is an emotion characterized by worry and physical symptoms of tension [1], and it is one of the most common and widespread mental health problems today [44]. The anxiety levels of university students are significantly higher than those of their non-student peers of the same age. A meta-analysis of 64 studies showed that the pooled prevalence of anxiety symptoms among university students was 39.0%, significantly higher than that of the general population [64]. Sudden public health emergencies can further exacerbate anxiety levels among college students. For example, during the COVID-19 pandemic, the general anxiety rate among Brazilian university students during remote instruction reached as high as 76.1% [95]. This indicates that anxiety is highly prevalent among diverse groups of college students worldwide [58]. Anxiety not only harms the mental health of university students but also causes a series of physiological and behavioral consequences. For instance, appearance anxiety, eating disorders, and social anxiety disorder are significantly correlated, and their interaction may further increase the risk of functional impairment [56]. Anxiety is also intertwined with sleep quality and physical activity, thereby negatively affecting both physical and mental health [75]. In addition, anxiety has numerous adverse effects on university students’ academic development [80, 109] and social behavior [19, 89]. The high incidence of anxiety among university students is closely tied to the multiple stressors they face. Jones et al. [58] pointed out that among many influencing factors, academic distress accounts for the largest proportion, followed by financial pressure and insufficient family and peer support. Therefore, exploring the pathways of anxiety among college students is of great practical significance for maintaining their mental health.

Physical activity (PA) refers to any bodily movement produced by skeletal muscle contractions that results in energy expenditure [10]. A substantial body of evidence consistently shows that PA helps alleviate anxiety in college students. At the cross-sectional level, Liu et al. [75] found a significant negative correlation between PA levels and social anxiety disorder based on a survey of 1,408 Chinese university freshmen. At the intervention and experimental level, 10 weeks of continuous exercise reduced trait anxiety in female university students [2], while cycling aerobic exercise, spontaneous relaxation training, and quiet rest all showed comparable anti-anxiety effects [34]. Aggregated evidence also supports this conclusion, with a meta-analysis by Lin and Gao [71] demonstrating that PA interventions exert positive effects on alleviating anxiety in college students. In the context of public health emergencies, the value of physical activity in reducing anxiety among college students becomes even more prominent. Xiang et al. [110] found that students who maintained high levels of physical activity during epidemic isolation experienced significantly milder anxiety symptoms, and home-based stretching and resistance training were negatively correlated with both anxiety and depression. A randomized controlled trial by Li et al. [64] further showed that 12 weeks of continuous Baduanjin practice reduced perceived COVID-19–related anxiety among college students and significantly improved their mental health. Overall, the relationship between PA and anxiety among college students is complex and multifaceted. Physical activity can directly alleviate symptoms and can also indirectly influence anxiety levels through various psychological, physiological, and social mechanisms. However, these potential pathways have not been fully elucidated and require further exploration to provide a basis for developing more targeted prevention and intervention measures.

Psychological resilience (PR) is defined as the psychological processes and behaviors that promote individual resources and protect them from the potential negative effects of stressors [33]. Research indicates that psychological resilience is negatively correlated with anxiety, depression, stress, and even suicidal ideation, highlighting its crucial role in protecting mental health [50, 81]. Longitudinal data collected during the COVID-19 pandemic further supports this association, demonstrating that higher levels of resilience predict reduced anxiety and stress over time [60]. Physical activity is considered one of the key ways to enhance psychological resilience [35, 113]. Dong et al. [27], using a cross-lagged panel model (CLPM), confirmed that a positive sports activity climate can improve students’ psychological resilience. Research by Huang et al. [46] similarly found a significant positive correlation between college students’ physical exercise and psychological resilience (*r* = 0.541). Notably, multiple studies have shown that psychological resilience plays a mediating role between physical activity and anxiety. Qin et al. [88] constructed a mediation model based on 997 university students and found that psychological resilience was significantly negatively correlated with anxiety and played a partial mediating role between physical exercise and anxiety. Liu et al. [72] confirmed in 1,380 university students that physical exercise and reduced anxiety were significantly indirectly linked through psychological resilience. Research by Jiang and Wang [54] indicated that psychological resilience played a significant partial mediating role between physical exercise and social physique anxiety, accounting for 27.11% of the total effect. Furthermore, Li et al. [66] found that during the home isolation period of the pandemic, psychological resilience also played a partial mediating role between physical exercise and negative emotions in university students, with the mediating effect contributing as much as 50.00%. However, some studies indicate that psychological resilience does not significantly mediate the relationship between physical activity and anxiety [87]. This suggests that existing research still has certain inconsistencies and limitations. Therefore, it is necessary to integrate multivariate evidence to further verify and deepen existing findings.

Social support (SS) refers to the recognition and affirmative feedback an individual receives through continuous interaction by establishing formal or informal relationships with social groups [9]. Among university students, a strong social support network is believed to effectively alleviate the stress and challenges encountered in daily life and promote mental health. Research has shown a significant negative correlation between social support and anxiety ([73]; Tham et al., 2020; [88]). Particularly during public health emergencies, a study conducted during COVID-19 indicated that social support plays an important role in alleviating anxiety symptoms, with a lower risk of anxiety significantly associated with higher levels of social support [70]. Furthermore, numerous studies have demonstrated that higher levels of social support are closely related to higher levels of physical activity. Research by Zhou et al. [122] found a significant positive correlation between physical activity and social support, a finding also supported by a meta-analysis conducted by Wang et al. [101], which showed a significant association between the two (*r* = 0.30). Meanwhile, Zhang et al. [116] pointed out that individuals with higher physical activity levels receive greater social support than those leading sedentary lifestyles. Physical activity not only directly improves individuals’ mental health but also promotes their physical and psychological development by enhancing social support [45]. It is noteworthy that multiple studies have shown that social support plays a significant mediating role between physical activity and anxiety among college students [25, 73, 120, 121]. However, to fully understand this pathway and synthesize the existing findings, more relevant studies need to be integrated through a systematic review.

Through a systematic review of existing literature, this study aims to explore the parallel mediating roles of psychological resilience and social support between physical activity and anxiety in college students and to propose a conceptual model based on the literature review. The model suggests that college students’ physical activity directly affects anxiety levels, with psychological resilience and social support serving as mediating variables. In other words, physical activity indirectly and negatively influences anxiety levels by enhancing psychological resilience and social support. In addition, research indicates that gender differences lead to variations in college students’ psychological resilience [13, 113] and social support [59, 101]. For example, when experiencing psychological distress, the psychological resilience of male college students is more strongly associated with their coping ability than with perceived social support, whereas female college students rely more heavily on perceived social support [117]. This may result in gender differences in the regulation of negative emotions. At the same time, participants who select different survey formats exhibit distinct sociodemographic characteristics, and differences are also found in the reported results collected through on-site sampling and online sampling [12]. On-site sampling can more accurately reflect actual conditions and improve the validity of research findings, but it typically requires considerable cost and time and may be prone to sampling bias in convenience samples [12]. Online sampling, on the other hand, provides great convenience for data collection and reduces researchers’ time and effort, but may lead to uncertainties in data quality, sampling bias [107], and self-selection bias [96]. These factors may affect the effectiveness of both pathways. Therefore, this study will conduct subgroup analyses on gender differences and sampling methods to further examine the impact of these moderating variables on the model pathways.

In summary, this study proposes the following hypotheses and employs meta-analytic structural equation modeling (MASEM) for verification, aiming to explore the impact of physical activity on anxiety among college students and to analyze the parallel mediating mechanisms of psychological resilience and social support in this relationship. It is expected to provide evidence-based support for the effective implementation of targeted prevention and intervention strategies to promote the mental health of college students.

- Hypothesis 1: Physical activity among college students has a negative impact on anxiety.
- Hypothesis 2: Psychological resilience plays a mediating role between physical activity and anxiety in college students.
- Hypothesis 3: Social support plays a mediating role between physical activity and anxiety in college students.
- Hypothesis 4: The proportion of females and the sampling method (on-site sampling vs. online sampling) moderate the model pathways.

## Methods

### Protocol registration

This study was conducted in strict accordance with the Preferred Reporting Items for Systematic Reviews and Meta-Analyses (PRISMA) guidelines [86]. The protocol was registered with the International Prospective Register of Systematic Reviews (PROSPERO) on August 19, 2025 (registration number: CRD420251129622).

### Inclusion and exclusion criteria

1. The study participants were university students (undergraduates or postgraduates, regardless of their mode of admission [11]). Studies involving non-university student samples were excluded.
2. The study content had to include the predictor variable (PA), mediator variable (PR or SS), and outcome variable (ANX), or studies including both mediator variables (PR and SS). Studies examining only a single variable were excluded.
3. Eligible study designs included observational studies and baseline data derived from randomized controlled trials (RCTs) and cohort studies. Studies that did not meet these criteria were excluded.
4. The study had to report sample size, Pearson correlation coefficients, or other convertible statistical indicators. For studies with two or more independent samples, each independent sample was coded separately. Studies from which effect sizes could not be extracted or converted were excluded.
5. The study had to use validated scales or measurement instruments, and their internal consistency (Cronbach’s α) could not be lower than 0.60; otherwise, they were excluded.
6. The study had to employ a quantitative research design. Therefore, review articles, qualitative studies, and studies not meeting these requirements were excluded.

### Search strategy

This study adopted a systematic review and meta-analytic structural equation modeling approach, strictly adhering to the PRISMA statement for literature retrieval and screening. To comprehensively and systematically collect relevant evidence, eight databases were searched: Cochrane Library, PsycINFO (via EBSCO), PubMed, Web of Science, Scopus, China National Knowledge Infrastructure (CNKI), VIP, and Wanfang Data. The search period extended from the inception of each database to July 24, 2025. No restrictions were imposed on language or publication type, and the reference lists of all included studies were screened to minimize the omission of relevant literature. The complete search strategy is provided in the supplementary materials (Supplementary Table 1).

### Study selection and data extraction

**Fig. 1:**
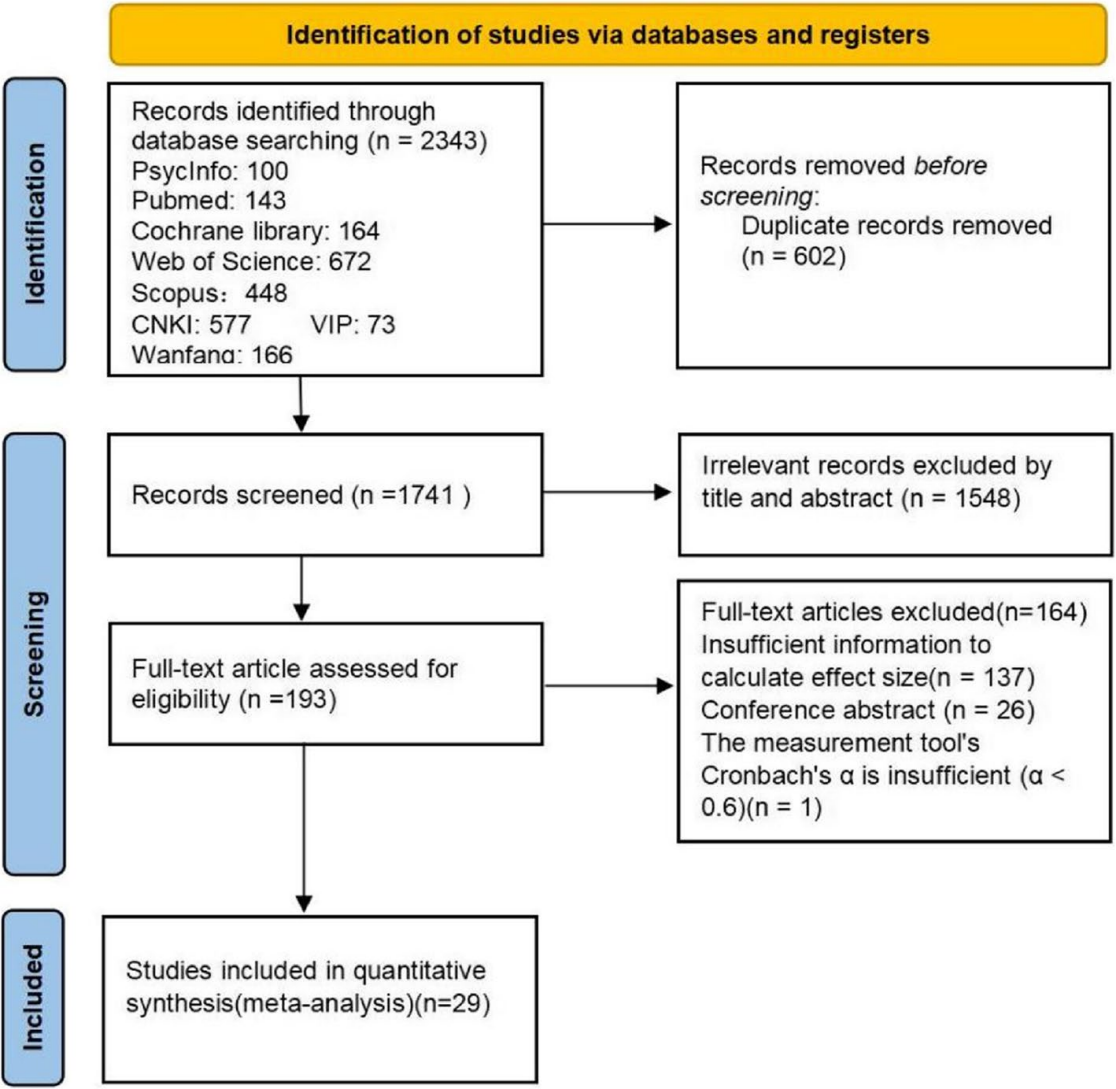
Study selection flow chart

Two reviewers (CG and JL) screened the literature using a combination of electronic database searches and manual supplementary searches, following the principle of “independent dual screening – consensus” to ensure scientific rigor and transparency. As illustrated in Fig. 1, 2343 records retrieved from various sources were first imported into EndNote 20 (Clarivate Analytics, Thomson Reuters Corporation) for automatic de-duplication. Remaining duplicates were then removed manually, resulting in 602 duplicate articles being excluded. The titles and abstracts of the remaining 1741 articles were independently reviewed by two reviewers (CG and JL) to exclude studies clearly inconsistent with the research topic. Disagreements at any stage were resolved through discussion, and if consensus could not be reached, a third reviewer (XL) was consulted. After full-text assessment of 193 potentially eligible articles, 26 conference abstracts (e.g., [97]), 137 studies from which effect sizes could not be calculated due to insufficient data (e.g., [8]), and 1 study with a Cronbach’s α < 0.60 for its measurement tool (e.g., [65]) were excluded. Ultimately, 29 studies were included.

Two reviewers (CG and JL) independently extracted data from all included studies using a standardized extraction form and cross-checked the information to ensure accuracy and completeness. Extracted data included: (1) study characteristics (first author, study design, sample country, publication year, sampling method); (2) demographic characteristics (sample size, age, percentage of females); (3) measurement instruments for the four variables and their Cronbach’s alpha values.

### Quality assessment

Two reviewers (CG and JL) independently assessed the methodological quality of the included studies; disagreements were resolved through discussion with a third reviewer (XL). Given the diversity of study designs, three standardized tools were selected for quality assessment. The quality of observational studies was assessed using the *Quality Assessment and Validity Tool for Correlational Studies* developed by Cicolini et al. [17]. This tool contains 13 items (14 points in total) across four dimensions: research design, sample characteristics, measurement tools, and statistical analysis. Twelve items are scored 0 (non-compliant) or 1 (compliant), and one item related to outcome measurement is scored 0 or 2. Based on total scores, studies were categorized as low quality (0–4), moderate quality (5–9), or high quality (10–14). This tool has also been used in previous MASEM studies (e.g., [104]). The quality of RCTs was assessed using the *Cochrane Collaboration’s Tool for Assessing Risk of Bias* recommended by Higgins et al. [42]. This tool consists of seven domains, including random sequence generation and allocation concealment, with each domain rated as “low risk,” “unclear risk,” or “high risk,” ensuring a systematic and objective evaluation of bias. The quality of cohort studies was assessed using the *Newcastle–Ottawa Scale* (NOS) developed by Wells et al. [105]. This scale evaluates risk of bias across three dimensions—Selection, Comparability, and Outcome—with a maximum score of 9 points. Based on established criteria, studies were classified as high quality (7–9 points), moderate quality (4–6 points), or low quality (< 4 points). This classification has also been applied in previous meta-analyses (e.g., [32]).

### Data synthesis and analysis

Meta-analytic structural equation modeling (MASEM) is a statistical approach that integrates meta-analysis with structural equation modeling (SEM) and can be used to test complex theoretical models involving multivariate relationships [124]. By synthesizing the correlation coefficient matrices from multiple primary studies, MASEM provides stronger evidence for the path relationships among variables and shows greater robustness in testing mediation effects [52]. This study uses the Comprehensive Meta-Analysis (CMA) software, follows the analytical framework of Hedges and Schmidt [49], and applies the Fisher z transformation to accurately estimate effect sizes.

The Pearson correlation coefficient (r) was adopted as the primary effect size to achieve a unified measurement of the degree of correlation among variables. For studies reporting only Spearman correlation coefficients (e.g., [88]), these were converted to Pearson’s r using the formula *r* = *2 sin(rs* × *π/6)* proposed by Myers and Sirois [82]. This conversion ensures the comparability of results across studies and represents a widely accepted practice in meta-analysis. In integrating multi-dimensional correlation coefficients, this study applied the algorithm by Olkin and Pratt [85] to compute the weighted average correlation, with results generated using a web tool (https://www.psychometrica.de/correlation.html). Furthermore, correlation coefficients were interpreted by absolute value following the recommendations of Hemphill [40]: weak correlation (|r|< 0.20), moderate correlation (0.20 ≤|r|≤ 0.30), and strong correlation (|r|> 0.30).

This study employed the two-stage structural equation modeling (TSSEM) method [16], using CMA and AMOS to implement MASEM. In the first stage, given the heterogeneity in sample and measurement characteristics across the original studies, correlation matrices were integrated using a random-effects model to estimate a pooled correlation matrix. Heterogeneity was assessed using Cochran’s Q test (*p* < 0.10 indicating heterogeneity) and the I^2^ statistic, where values of 0–25%, 25–50%, 50–75%, and > 75% indicate insignificant, low, moderate, and high heterogeneity, respectively [41]. The second stage involved fitting a structural equation model based on the pooled correlation matrix [15]. The harmonic mean of the included sample sizes was used to determine the significance of the estimated coefficients [7], and the bootstrap method was applied to test mediating effects. Model fit was first assessed using the χ^2^ test, with the χ^2^ value adjusted using the Bollen–Stine bootstrap method to reduce the risk of overestimating model misfit under large samples [5, 6]. Additionally, multiple indices were used to evaluate model fit, including χ^2^/DF, AGFI, SRMR, TLI, CFI, IFI, and Hoelter’s N [43, 51]. Moreover, because meta-analyses often include the same latent variable measured by different scales, directly integrating covariance matrices may lead to methodological bias [106]. Therefore, this study used a pooled correlation matrix rather than a covariance matrix for analysis [4, 14].

Moderation analysis was conducted to examine whether other variables influenced the model’s path coefficients. This study used the proportion of females and the sampling method as moderating variables. Due to the limited number of studies employing a mixed on-site and online sampling approach—and considering the potential limitations of online surveys in response rates and reliability as noted by Nulty et al. [83]—the sampling method was categorized into two subgroups: on-site sampling and online sampling. All categorical variables were first dummy-coded, followed by moderation analysis on the second-stage path coefficients using a one-stage meta-analytic structural equation model (OSMASEM) [53]. The significance of moderating effects was determined by comparing parameter estimates across models,when *p* ≤ 0.05, moderation was considered significant, and further comparisons of path coefficients between groups were conducted.

To examine the robustness of the results, sensitivity analyses and publication bias tests were performed to evaluate potential bias and the influence of outliers. First, a leave-one-out strategy was used for sensitivity analysis. Second, because converting Spearman to Pearson correlation coefficients may introduce estimation bias, a specific sensitivity analysis was conducted for this conversion. Specifically, pooled results based solely on original Pearson coefficients (i.e., unconverted data) were compared with mixed pooled results that included Spearman-converted data. Differences in the main effect sizes and changes in heterogeneity (I^2^) were examined to assess the impact of the conversion. Regarding publication bias, following Wen et al. [104], funnel plots were generated to visually inspect symmetry when nine or more studies were available for a given path. Additionally, Fail-safe N and Egger’s linear regression test were used for statistical assessment [30, 90]. When discrepancies were observed among results, the trim-and-fill method was applied for further verification [29].

## Results

### Study characteristics

Table 1 summarizes the main characteristics and results of the 29 included studies. Among all studies, 27 adopted a cross-sectional design, 1 was a randomized controlled trial (RCT), and 1 was a prospective cohort study. Since Margraf et al. [79] simultaneously reported two independent samples from Germany (*n* = 591, online sampling) and China (*n* = 8831, on-site sampling), this study coded them as two separate study units. Therefore, a total of 30 sample units were included, and 90 effect sizes were extracted (see Supplementary Table 2).These studies were conducted across various countries and regions, including China, Turkey, South Africa, the United States, and Germany. The total sample size was 36,159 university students, with individual study sample sizes ranging from 72 to 8,831. The age of participants ranged from 18 to 29 years. Regarding sampling methods, 12 studies (40.0%) used on-site sampling, 7 studies (23.3%) used online sampling, and 7 studies (23.3%) used mixed online and on-site sampling. Another 4 studies (13.3%) did not report specific sampling methods. The proportion of female participants ranged from 27.78% to 68.98%. Quality assessment results indicated that all included correlational studies achieved at least a moderate quality rating. Specifically, quality scores for observational studies ranged from 6 to 13, with a mean of 10.48. Among these, 5 studies were classified as moderate quality and 22 as high quality (see Supplementary Table 3). For the single included RCT, the risk of bias assessment showed an overall “low risk” (Supplementary Table 4). The prospective cohort study received a score of 5, corresponding to moderate quality according to the established criteria (Supplementary Table 5).

**Table 1:** Characteristics of studies included in the meta-analysis

Study name	Study design	Sample size	Age (year)	Area	Sampling method	Female (%)	Include variable
Lian [67]	Cross-sectional	1130	Nr	China	On-site	48.8	PA,PR,SS
Cui [20]	Cross-sectional	824	Nr	China	On-site	51.9	PA,PR,SS
Guo [39]	Cross-sectional	988	Nr	China	On-site + Online	47.57	PA,PR,ANX
Cui et al. [21]	Cross-sectional	640	Nr	China	Nr	60.5	PA,PR,ANX
Liang [69]	Cross-sectional	698	Nr	China	Online	67.91	PA,SS,ANX
Liu [76]	Cross-sectional	1408	20.12 ± 1.21	China	On-site	48.3	PA,PR,ANX
Liao et al. [68]	Cross-sectional	716	Nr	China	On-site + Online	40.22	PA,PR,ANX
Wei [103]	Cross-sectional	367	Nr	China	On-site	68.9	PA,PR,ANX
Li Jinying [61]	Cross-sectional	1532	Nr	China	On-site + Online	47	PA,PR,SS
Ding et al. [26]	Cross-sectional	1838	Nr	China	On-site + Online	56.5	PA,PR,SS
Li Pengcheng [62]	Cross-sectional	476	Nr	China	On-site + Online	53.8	PA,PR,ANX
Wang Zhifeng et al. [102]	Cross-sectional	2642	Nr	China	Online	53.22	PA,PR,ANX
Zhu and Liu [123]	Cross-sectional	325	Nr	China	Nr	57.54	PA,PR,ANX
Xiu [112]	Randomized trial data	72	Nr	China	On-site	27.78	PA,PR,ANX
Demir and Barut [24]	Cross-sectional	360	20.73 ± 1.211	Turkey	On-site	56.7	PA,PR,ANX
Deng and Wang [25]	Cross-sectional	874	Nr	China	On-site	44.85	PA,SS,ANX
Jiang et al. [55]	Cross-sectional	1071	Nr	China	Nr	49.3	PA,PR,ANX
Jiang and Wang [54]	Cross-sectional	1177	19.12 ± 1.21	China	On-site + Online	46.2	PA,PR,ANX
Johannes et al. [57]	Cross-sectional	534	21.11 ± 2.71	South Africa	On-site + Online	53.6	PA,SS,ANX
Liu, S. et al. [74]	Cross-sectional	1658	Nr	China	Nr	65.62	PA,PR,ANX
Liu and Shi [73]	Cross-sectional	577	Nr	China	On-site	68.98	PA,SS,ANX
Liu et al. [72]	Cross-sectional	1380	Nr	China	On-site	44	PA,PR,ANX
Maier and James [78]	Cross-sectional	859	18.71 ± 1.22	USA	On-site	60	PA,SS,ANX
Margraf et al. [79]	Longitudinal	591 + 8831	18–29	Germany + China	On-site + Online	61.11	PR,SS,ANX
Qin et al. [88]	Cross-sectional	997	Nr	China	Online	43.63	PA,PR,SS,ANX
Wu et al. [108]	Cross-sectional	590	19.67 ± 1.48	China	On-site	53.9	PA,PR,ANX
Yue et al. [115]	Cross-sectional	2217	Nr	China	Online	68.34	PA,PR,ANX
Zhao, H. et al. [118, 119]	Cross-sectional	534	Nr	China	Online	50.75	PA,PR,ANX
Zheng et al. [120, 121]	Cross-sectional	325	Nr	China	Online	49.23	PA,PR,SS,ANX

*Nr* not reported, *PA* Physical Activity, *PR* Psychological Resilience, *SS* Social Support, *ANX* Anxiety

### The first-stage analysis

Table 2 presents the results of six sets of pairwise correlation meta-analyses based on the random-effects model. All effect sizes were statistically significant (*p* < 0.001), with absolute correlation coefficients ranging from 0.247 to 0.443. Among them, the PR–ANX correlation was the strongest (*r* = –0.443, 95% CI: –0.505, –0.376), whereas the PA–SS correlation was the weakest (*r* = 0.247, 95% CI: 0.152, 0.337). Overall, except for PA–SS, which reached a moderate level, the remaining five correlation pairs demonstrated strong associations.

**Table 2:** Summary of bivariate correlations estimated by random-effects meta-analysis (TSSEM-Stage 1)

Pairwise relationships	K	N	Effect Size(r)	P	95% CI		Heterogeneity		FSN	Egger's P
					Lower	Upper	Q-value	I2		
PA-ANX	21	18374	−0.368	0.000	−0.484	−0.240	1867.308***	98%	653	0.849
PA-PR	22	22907	0.375	0.000	0.286	0.458	1212.763***	98%	4000	0.263
PA-SS	10	9654	0.247	0.000	0.152	0.337	212.257***	95%	1322	0.184
PR-ANX	20	25148	−0.443	0.000	−0.505	−0.376	690.568***	97%	8878	0.125
PR-SS	8	16068	0.418	0.000	0.339	0.492	184.819***	96%	4346	0.058
SS-ANX	9	14286	−0.322	0.000	−0.403	−0.236	170.301***	95%	1976	0.002

K = number of effect sizes; N = cumulative sample size; r = pooled correlation coefficient, *CI* confidence interval, *FSN* fail-safe number

^***=^*p* <.001

To assess the robustness of the results, this study conducted heterogeneity tests and publication bias analyses. The Q-values for all relationships were significant (*p* < 0.001), indicating substantial heterogeneity among studies. Similarly, the I^2^ values of paired correlations ranged from 95 to 98%, reflecting high heterogeneity. Funnel plots were generated for five relationships: physical activity with psychological resilience, social support, and anxiety, as well as psychological resilience and social support with anxiety. Visual inspection showed that four funnel plots were approximately symmetric (Figures Supplementary 1–4), while the funnel plot for the relationship between social support and anxiety displayed some asymmetry (Figure Supplementary 5). Regarding statistical assessment (Table 2), the Fail-Safe Number (FSN) test suggested that none of the relationships were affected by substantial publication bias. However, Egger’s linear regression test indicated a statistically significant risk of publication bias for the social support–anxiety relationship (*p* = 0.002), whereas no significant bias was detected for the other relationships (*p* > 0.05). Given this inconsistency, the trim-and-fill method was applied for further verification. The results showed that no studies needed to be imputed (i.e., number of imputed studies = 0), suggesting that the asymmetry was not attributable to missing studies.

### The second stage analysis

**Fig. 2:**
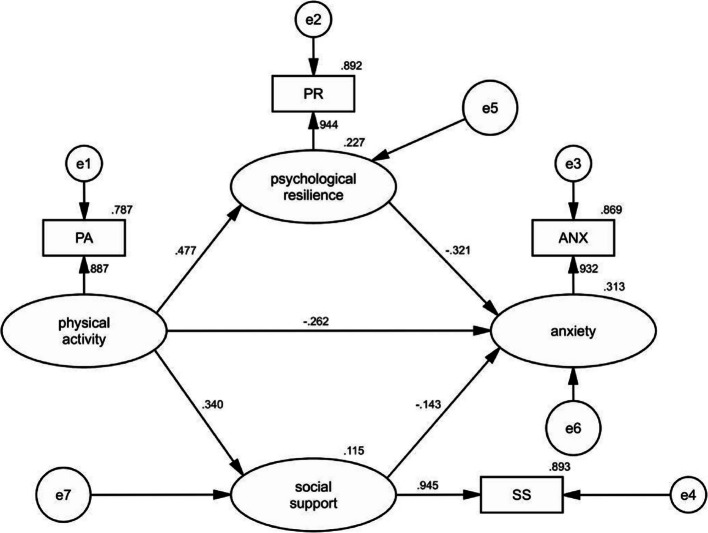
Meta-analytic structural equation model for this study

Tables 3 and 4, and Fig. 2 present the results of the second phase of the TSSEM analysis. First, the correlation matrix obtained from the first stage of TSSEM was imported into AMOS. Measurement errors were fixed at 1 − α, and factor loadings were set to the square root of alpha, where alpha represents the mean reliability of each variable (Supplementary Table 6). The sample size was determined using the harmonic mean (*n* = 16,057) [100], after which the maximum likelihood estimation method was applied to estimate the model paths [15]. The direct effects in Table 3 show that physical activity has a significant positive effect on psychological resilience (Std = 0.477, *P* ≤ 0.001) and social support (Std = 0.340, *P* ≤ 0.001); whereas physical activity (Std = − 0.262, *P* ≤ 0.001), psychological resilience (Std = − 0.321, *P* ≤ 0.001), and social support (Std = − 0.143, *P* ≤ 0.001) exhibit negative effects on anxiety. Furthermore, the fit indices of the structural equation model indicate good model fit: Bollen–Stine χ^2^ = 1.014, Normed Chi-square (χ^2^/DF) = 1.014, AGFI = 0.999, SRMR = 0.052, TLI = 1.000, CFI = 1.000, IFI = 1.000, and Hoelter’s *N* = 18,131.296, demonstrating that the model possesses high statistical reliability and explanatory power.

**Table 3:** Model path coefficient analysis

Path	Std	S.E	95%CI	Z-value	*P*-value
Physical Activity → Psychological Resilience	0.477	0.008	0.460,0.492	59.625	***
Physical Activity → Social Support	0.340	0.009	0.322,0.357	37.778	***
Physical Activity → Anxiety	−0.262	0.010	−0.281,−0.243	−26.200	***
Psychological Resilience → Anxiety	−0.321	0.009	−0.339,−0.303	−35.667	***
Social Support → Anxiety	−0.143	0.009	−0.160,−0.125	−15.889	***
Model fit**:** Bollen-Stine χ^2^ = 1.014, Normed Chi-sqr (χ^2^/DF) = 1.014,AGFI = 0.999, SRMR = 0.052, TLI = 1.000,CFI = 1.000, IFI = 1.000, Hoelter's *N* = 18131.296					

*Std*. standardized coefficient, *S.E*. standard error, *CI* confidence interval, ***:*P* ≤ 0.001, χ^2^ = Chi-square, *DF* Degrees of Freedom, *AGFI* Adjusted Goodness of Fit Index, *SRMR* Standardized Root Mean Square Residual, *TLI* Tucker–Lewis index, *CFI* Comparative Fit Index, *IFI* Incremental Fit Index, Hoelter's N = Hoelter's Critical N

**Table 4:** Mediation effect test

Path relationship	Point estimate	Product 0f coefficient		Bootstrapping			
				Bias-corrected 95% CI		Percentile 95% CI	
		SE	Z-value	Lower	Upper	Lower	Upper
Indirect Effects							
PA → PR → ANX	−0.152	0.005	−30.400	−0.163	−0.142	−0.163	−0.142
PA → SS → ANX	−0.048	0.003	−16.000	−0.055	−0.042	−0.055	−0.042
Total Effects							
PA → ANX	−0.461	0.009	−51.222	−0.479	−0.443	−0.479	−0.443

In the mediating effect test, this study examined the mediating mechanisms underlying the influence of physical activity on anxiety using 5000 bootstrap samples under a 95% confidence interval. As shown in Table 4, increases in physical activity significantly reduce anxiety, with a total effect of − 0.461 (95% CI: − 0.479, − 0.443). Psychological resilience (β = − 0.152, 95% CI: − 0.163, − 0.142) and social support (β = − 0.048, 95% CI: − 0.055, − 0.042) play partial mediating roles between physical activity and anxiety. Although the mediating effect of social support is smaller, it remains statistically significant (Z = − 16.000).

### Moderator analysis

A subgroup analysis was conducted using OSMASEM based on the proportion of females and the sampling method. The results of the model path comparisons indicate that the proportion of females significantly moderates all five paths (*P* < 0.001), whereas the sampling method significantly moderates three paths: physical activity → social support, physical activity → anxiety, and social support → anxiety (*P* < 0.001). The between-group comparison in Table 5 further shows that when the proportion of females is ≤ 50%, the effects of physical activity on psychological resilience (β = 0.510) and social support (β = 0.374), as well as the effects of psychological resilience (β = − 0.398) and social support (β = − 0.206) on anxiety, are all stronger than the corresponding paths when the proportion of females is > 50% (β = 0.439; β = 0.303; β = − 0.268; β = − 0.118). However, when the proportion of females is > 50%, the effect of physical activity on anxiety (β = − 0.297) is greater than the same path when the proportion of females is ≤ 50% (β = − 0.196). Regarding sampling method, the effects of physical activity on social support (β = 0.448) and social support on anxiety (β = − 0.240) in online sampling studies are greater than in on-site sampling studies (β = 0.349; β = − 0.037). It is noteworthy that the effect of physical activity on anxiety (β = − 0.346) in on-site sampling studies is stronger than the corresponding path in online sampling studies (β = − 0.215).

**Table 5:** The significance of model path comparisons(P) and the standardized path coefficients of parameter estimates in subgroup analysis

Variable	Path a	P	Path b	P	Path c	P	Path d	P	Path e	P
Proportion of women										
≤ 50%	0.510	0.000	−0.398	0.000	0.374	0.000	−0.206	0.000	−0.196	0.000
> 50%	0.439		−0.268		0.303		−0.118		−0.297	
Sampling methods										
On-site	0.551	0.866	−0.287	0.198	0.349	0.000	−0.037	0.000	−0.346	0.000
Online	0.552		−0.252		0.448		−0.240		−0.215	

Path a: Physical activity → Psychological Resilience; path b: Psychological Resilience → Anxiety; path c: Physical activity → Social Support; path d: Social Support → Anxiety; path e: Physical activity → Anxiety

### Sensitivity analysis

This study conducted a sensitivity analysis on six pairs of variable relationships by sequentially removing individual studies and recalculating the pooled correlation coefficients. The results showed that, regardless of which study was removed, the direction of the pooled correlation coefficients for all six pathways remained unchanged, indicating that the analytical results are highly robust (Supplementary Figs. 6–11). Furthermore, the results of the sensitivity analysis regarding data conversion (Supplementary Table 7) showed that the pooled effect sizes and heterogeneity levels (I^2^) were largely consistent between the subset of studies containing only original Pearson correlation coefficients and the mixed dataset incorporating Spearman-converted data. This suggests that the Spearman conversion had minimal influence on the results, thereby further demonstrating the high robustness of the analytical findings.

## Discussion

### Main findings

This study included 29 articles, with a total sample size of 36,159 participants. Using the MASEM method, a correlation coefficient matrix with 90 effect sizes was constructed to examine the relationships among PA, PR, SS, and ANX and their potential mediating effects. Although existing literature reports inconsistencies in conclusions regarding the internal pathways, this study provides new empirical evidence. The results indicated a significant correlation between physical activity and anxiety among university students (*P* < 0.001), and all path coefficients for PA, PR, SS, and ANX were significant (*P* < 0.001). At the same time, the findings further revealed that psychological resilience and social support play a significant mediating role in the relationship between physical activity and anxiety (*P* < 0.001). Subgroup analysis results showed that the proportion of females in the sample and the sampling method moderated the model pathways. Regarding the inconsistency in publication bias test results for the relationship between social support and anxiety, this may be attributable to the inherent differences in sensitivity and emphasis among the statistical methods. Specifically, funnel plots and Egger’s test are particularly sensitive to small-study effects or high between-study heterogeneity [30], thus, the observed statistical significance may reflect heterogeneity rather than publication bias alone. In contrast, the Fail-safe N and trim-and-fill methods focus more on assessing whether potential unpublished negative studies are sufficient to overturn the current pooled effect [29]. The present analysis indicates that, unless an extremely large number of unpublished null studies exist, the effect remains robust (as the FSN far exceeds the critical threshold). Moreover, the trim-and-fill results (imputing 0 studies) further corroborate the reliability of the effect. In summary, although a potential risk of small-study bias was observed for this pathway, it did not substantially undermine the validity of the core conclusion. Furthermore, no significant indications of publication bias were detected for the other relationships examined in this study. Combined with the results of the sensitivity analyses, the overall findings demonstrate high robustness.

### The relationship between physical activity and anxiety

This study examined the negative impact of physical activity on anxiety, and the results are consistent with existing literature, showing that physical activity can alleviate anxiety among university students, thereby confirming hypothesis H1. Multiple studies have demonstrated that physical activity significantly reduces anxiety levels in university students [118, 119], particularly social anxiety [47]. A study by Dong et al. [28] indicated that different exercise intensities exert varying degrees of influence on anxiety, with high-intensity physical activity directly affecting trait anxiety levels in university students. Furthermore, a meta-analysis conducted by Lin and Gao [71] confirmed the positive effect of physical activity interventions on alleviating anxiety in university students, and aerobic exercise was identified as the optimal intervention approach. Physical exercise can accelerate blood circulation in the brain and throughout the body, promoting the development of intelligence and creativity. At the same time, it can divert attention, enabling individuals to break free from troubling thoughts [48]. Moreover, physical exercise significantly improves serotonin, BDNF, and cortisol levels in university students, thereby alleviating their anxiety [120, 121]. These research findings align with the present study. However, this study further validated the negative relationship between physical activity and anxiety by constructing a model and expanded the explanatory boundaries of this relationship, providing evidence-based support for future intervention strategies.

### The mediating mechanisms of psychological resilience and social support

This study further explored the mediating role of psychological resilience in the relationship between physical activity and anxiety. The results indicate that physical activity positively predicts psychological resilience in university students and indirectly affects their anxiety levels through psychological resilience. In other words, psychological resilience plays a partial mediating role between physical activity and anxiety among university students, thus verifying Hypothesis H2. As shown in the study by Li et al. [65], psychological resilience mediates the relationship between physical activity and anxiety in university students, and increasing physical activity levels can reduce social anxiety by enhancing psychological resilience. The study by Zhao et al. [118, 119] further confirmed that physical exercise positively predicts psychological resilience in university students and negatively predicts social anxiety, with psychological resilience serving as a mediating mechanism between physical exercise and social anxiety. Physical exercise can enhance an individual's psychological resilience, and psychological resilience—as a tenacious psychological quality—can be transferred into daily life and academic contexts, helping university students better cope with difficulties and challenges, maintain a stable psychological state, and consequently reduce anxiety (Cui et al., 2023). A study on deaf and hard-of-hearing university students further indicates that psychological resilience is a significant factor in managing interpersonal relationships and coping with social anxiety, and that it can be strengthened through physical exercise. Greater engagement in physical activity may lead to increased self-confidence, thereby enhancing the psychological resilience of deaf and hard-of-hearing university students and reducing their social anxiety. This progressive relationship creates favorable conditions for psychological resilience to function as a mediating variable [123]. Especially during the COVID-19 period, psychological resilience played an important buffering role, enabling individuals to better “rebound,” maintain physical and mental health, and reduce anxiety caused by stressful events [111]. Therefore, universities can actively organize physical activity programs to enhance students’ psychological resilience, which in turn can reduce anxiety and promote healthy physical and mental development.

This study also found that social support played a partial mediating role between physical activity and anxiety, a result consistent with existing research, thereby verifying Hypothesis H3. Studies have shown that physical exercise can effectively reduce anxiety, with social support significantly mediating the relationship between physical exercise and anxiety [73, 120, 121]. The study by Deng and Wang [25] also pointed out that in addressing social anxiety among university students, physical activity can not only directly reduce anxiety but also indirectly alleviate it by increasing levels of social support. This suggests that social support indeed exerts a buffering effect on anxiety. As a coping resource, social support can help individuals reduce or more easily tolerate stress [99], and it plays a buffering role between the subjective experience of stress and the onset of illness, mitigating the negative impact of stressful events on physical and mental well-being and helping maintain and improve overall health [36]. At the same time, through social interactions embedded in physical activity, individuals can perceive and receive more understanding, assistance, and support from family and friends, thereby enhancing positive internal cognition, alleviating psychological stress, and reducing the impact of negative experiences [67]. Therefore, universities can provide students with more opportunities to participate in physical activities and strengthen mutual support systems through social events, which may effectively alleviate anxiety and promote the physical and mental health of university students.

### Impact of moderating factors

The results of the subgroup analysis indicated that the proportion of female participants in the sample and the sampling method significantly moderated various paths in the model, thus supporting hypothesis H4. When the proportion of females exceeds 50%, the effects of physical activity on psychological resilience and social support, as well as the effects of psychological resilience and social support on anxiety, are all weakened. First, studies have shown significant gender differences in adherence to physical activity recommendations, particularly in high-intensity activities, where males outperform females, and male participants demonstrate higher levels of psychological resilience compared with female participants [114]. Meanwhile, as physical activity levels increase, female university students typically perceive higher levels of social support than male students [94]. This result is inconsistent with the findings of the present study, and may be influenced by beliefs, expectations, and arguments that underlie exercise motivation (i.e., attitudes), which may differ across gender [84]. Secondly, research indicates that women are more prone to social anxiety disorder than men [98]. A review study reported that women exhibit higher levels of social anxiety than men, which may be related to the anxiety-induced enhancement of female parasympathetic nervous system reactivity. At the same time, studies show that men are more likely than women to seek treatment for social anxiety disorder [3]. Furthermore, women are more negatively affected during interpersonal interactions, and when they feel misunderstood, their decline in daily life satisfaction is greater than that observed in men, and may be accompanied by more physical symptoms [77]. Additionally, the present study found that when the proportion of females exceeds 50%, the effect of physical activity on anxiety is strengthened, possibly because females are more concerned with body image issues. A study on body image among male and female university students indicated that females exhibit a higher proportion of body image concerns and pay greater attention to body image [31]. In contrast, young women who are highly concerned with their body shape and appearance may experience more pronounced emotional improvement and stress relief when engaging in physical activity, potentially promoting mental health more effectively [38].

This study also found that the sampling method significantly moderated specific paths within the model. However, these results should be interpreted with caution due to the limitations associated with the relatively small sample size in the online sampling subgroup (*N* = 3,095). Despite this limitation, the observed trends retain theoretical significance. In the analysis of sampling methods, online sampling studies showed that the effects of physical activity on social support and the effects of social support on anxiety were both stronger than those in on-site sampling. By contrast, in on-site sampling studies, the effect of physical activity on anxiety was greater than that observed in online sampling. These differences may be related to biases in the sampled population, as well as response rates and participation levels during data collection. Studies have shown that offline and online populations differ across multiple dimensions such as age, gender, and race [18], and selection bias in online surveys leads many adjusted estimates to still differ significantly from target estimates based on complete samples [92]. However, during the COVID-19 pandemic, the number of online studies using rating scales increased substantially, and research methods shifted heavily from in-person to online sampling [22]. A study by Grewenig et al. [37] indicated that although online sampling covers internet users who participate in surveys, it typically excludes a non-negligible proportion of the population that does not use the internet. Since offline populations may differ from online populations in preferences, beliefs, or sociodemographic characteristics, significant response differences between the two groups are indeed likely, and are mainly driven by survey mode effects. Additionally, vigilance toward internet use may also result in distinct pathways. The study by Sabah et al. [91] examined differences in social sensitivity between participants in online and offline environments and found that higher levels of online vigilance increased social sensitivity, thereby heightening vulnerability to psychological distress and negatively affecting mental health. Meanwhile, research by Seabrook et al. [93] suggests that positive face-to-face interactions may serve as a buffer to mitigate the negative effects of heightened online social sensitivity. Although findings from the online subgroup should be interpreted cautiously, they nonetheless provide valuable insights into how different data collection methods may influence the relationships among variables. Consequently, researchers should carefully consider the potential impact of sampling method when making methodological decisions.

Furthermore, all effect sizes in this study exhibited extremely high heterogeneity (I^2^ = 95–98%). Due to insufficient sample sizes across specific dimensions (e.g., measurement tools, cultural backgrounds) in the included literature, we were unable to conduct more comprehensive meta-regression or subgroup analyses. According to Higgins et al. [41], statistical heterogeneity in meta-analysis is often unavoidable due to the diversity of measurement tools and methodologies. Although we attempted to account for some sources of heterogeneity through subgroup analyses (targeting sampling methods and the proportion of female participants), the explanatory power remains limited. Consequently, we propose that the potential sources of heterogeneity are primarily concentrated in the following areas: First, differences in measurement tools represent a common source of heterogeneity. Although the included studies used validated scales, variations in construct operationalization, dimensionality, and psychometric focus resulted in inconsistent measurement criteria for physical activity, anxiety, resilience, and social support, thereby contributing to discrepancies in effect estimates. Secondary sources of heterogeneity may stem from sociocultural and regional disparities [23]. The included studies encompass diverse geographic regions—including China, Turkey, South Africa, the United States, and Germany—where distinct cultural backgrounds significantly shape university students' cognitive and behavioral patterns regarding mental health and physical activity. For instance, in collectivist cultures, the mechanism of “social support” may be more pronounced, as physical activities are often organized within collective or class-based structures. Conversely, in individualistic cultures, the pathway of "psychological resilience" may exert a more dominant influence. These cultural variations inevitably contribute to the observed statistical heterogeneity.Thirdly, variability in PA modalities constitutes another potential source of heterogeneity. Methodological inconsistencies in defining and measuring PA are compounded by the wide spectrum of activities involved, ranging from aerobic and team sports to mind–body exercises. These distinct modalities likely modulate anxiety through differing mechanisms: team sports may alleviate anxiety primarily by fostering social support networks, whereas mind–body exercises may operate by directly enhancing emotion regulation and resilience. Since the majority of included studies did not stratify by exercise type, amalgamating these diverse interventions into a single "physical activity" variable likely introduces significant statistical heterogeneity. Furthermore, variations in exercise intensity, frequency, and duration are also critical confounding factors.Finally, temporal variations in sampling contexts (i.e., pre-, during, and post-COVID-19) may further exacerbate between-study heterogeneity. The inclusion of studies conducted during the COVID-19 pandemic introduces unique stressors—such as lockdowns, remote learning, and health-related anxieties—that caused baseline anxiety levels to deviate sharply from pre-pandemic norms. Concurrently, the nature of PA shifted during lockdowns, notably transitioning from team-based sports to solitary home-based workouts. Consequently, the correlation between PA and anxiety documented during the pandemic may diverge from patterns observed in non-pandemic periods, potentially intensifying the overall heterogeneity. Despite the high heterogeneity, the MASEM approach remains suitable for estimating overall trends when handling such datasets. Moreover, this study employed a random-effects model combined with Bootstrap corrections, thereby ensuring the robustness and reliability of the findings.

### Strengths and limitations

This study comprehensively searched eight Chinese and English databases and integrated the results of 29 relevant studies with a total sample size of 36,159 participants, thereby providing a more reliable evidence base for the research. The study employed the TSSEM method to conduct MASEM, exploring the interrelationships among physical activity, psychological resilience, social support, and anxiety. It also confirmed the mediating roles of psychological resilience and social support in the relationship between physical activity and anxiety, providing a theoretical basis for mental health interventions targeting university students. Additionally, subgroup analyses on gender ratio and sampling methods revealed the significant moderating effects of these factors on the model’s pathways, further enhancing the depth and breadth of the research. Meanwhile, the results of the sensitivity analyses and multiple publication bias tests indicated that the findings are robust and not substantially affected by publication bias, and the model demonstrated good fit indices, underscoring the statistical reliability of the research and strengthening the study’s credibility.

Despite these strengths, several limitations should be acknowledged. First, the included studies were predominantly cross-sectional, and although one longitudinal study was incorporated, it was insufficient to examine the longitudinal dynamic relationships among variables. Therefore, the data could only assess associations rather than establish definitive causal relationships. Secondly, many included studies relied on participants’ self-reports, which may introduce social desirability bias or recall bias, thereby affecting the accuracy and objectivity of the results. Third, potential biases may arise from data conversion. Some studies did not directly report Pearson correlation coefficients. Although the present study strictly followed standard meta-analytic procedures for coefficient conversion, such conversions are inherently approximate. Issues associated with data distribution or the presence of outliers may lead to inaccuracies or oversimplification during the conversion process, thereby affecting the precision of the effect sizes. Furthermore, given that Chinese samples comprised a large proportion of the included studies, the findings may be more representative of university student populations within a Chinese cultural context. Finally, limitations concerning subgroup sample size and interpretability should be noted. Although subgroup analyses were conducted based on gender and sampling method, the sample size for certain subgroups (e.g., the online sampling group) was relatively small. This imbalance may limit the stability of parameter estimates within these subgroups, potentially masking other underlying moderating effects and restricting a more comprehensive examination of structural stability across different models. Additionally, although subgroup findings were statistically significant, their practical implications may be limited; thus, these conclusions should be interpreted with caution.

### Practice and future directions

Research indicates that physical activity can not only directly alleviate anxiety in university students but also indirectly reduce anxiety by enhancing psychological resilience and social support, thereby providing evidence-based support for mental health interventions. Therefore, universities should actively offer sports courses and encourage students to participate in physical activity to improve psychological resilience and social support, thus reducing anxiety [25, 64, 72]. At the same time, universities should regularly organize fitness activities, group sports, and other programs, implement psychological resilience training, and establish student sports interest groups or clubs, emphasizing the importance of resilience and social support in maintaining students’ mental health. Furthermore, targeted prevention and intervention measures should be introduced for different groups to more effectively promote the physical and mental well-being of university students.

To address the aforementioned limitations, several directions may guide future research. First, we suggest that future studies adopt longitudinal designs to explore the causal relationships among the four variables, thereby clarifying their temporal trends and dynamic interactions. Second, future research could combine objective measurement tools with subjective assessments to reduce bias associated with self-reported data. Moreover, future studies should consistently report correlation analysis data, enabling more comprehensive meta-analytic summaries using TSSEM [16]. Furthermore, research should include more studies across diverse cultural backgrounds to facilitate cross-cultural comparisons. Finally, future studies should adopt rigorous control designs and conduct more complex multifactorial analyses to deepen the understanding of the diverse factors influencing anxiety among university students, thus enabling the development of more targeted preventive and intervention strategies.

## Conclusion

This study systematically examined the interrelationships among physical activity, psychological resilience, social support, and anxiety in university students. Using the MASEM method, it evaluated the impact of physical activity on anxiety while analyzing the parallel mediating roles of psychological resilience and social support. The results demonstrate that increasing physical activity levels can effectively reduce anxiety among university students, and that psychological resilience and social support serve as significant mediators in this relationship. Furthermore, the study found that the proportion of female participants and the sampling method exert moderating effects on the model. Future research should emphasize longitudinal designs to explore the dynamic changes and causal relationships among variables, thereby refining the theoretical framework of university students’ mental health, providing more effective support strategies, and promoting students’ overall physical and psychological development.

## Supplementary Information

Supplementary Material 1.

## Data Availability

All data generated or analyzed during this study are included in this published article and its supplementary information files.
