# Supplementary material for: The influence pathways of physical activity on anxiety in university students: a systematic review and meta-analytic structural equation modeling study based on psychological resilience and social support
Source: BMC Psychol. 2026 Jan 12;14:193. doi: 10.1186/s40359-025-03915-2 (PMC12888664; doi:10.1186/s40359-025-03915-2)
Supplement: Supplementary file 1 — Supplementary Material 1. [file 40359_2025_3915_MOESM1_ESM.docx]

**Contents of supplementary appendix**

1 The search strategy and results1

**2 Other characteristics of studies included in the meta-analysis5**

3 Quality Assessment for each study individually10

4 Measurement error and factor loading(TSSEM-Stage 2)16

5The results of funnel plot18

Funnel plot of the correlation between physical activity and anxiety18

Funnel plot of the correlation between physical activity and psychological resilience19

Funnel plot of the correlation between physical activity and social support20

Funnel plot of the correlation between psychological resilience and anxiety.21

Funnel plot of the correlation between social support and anxiety.22

6 The results of sensitivity analysis23

Sensitivity analysis of the correlation between physical activity and anxiety.23

Sensitivity analysis of the correlation between physical activity and psychological resilience24

Sensitivity analysis of the correlation between physical activity and social support.25

Sensitivity analysis of the correlation between psychological resilience and anxiety.26

Sensitivity analysis of the correlation between social support and anxiety27

**Sensitivity analysis of the correlation between psychological resilience and social support28**

**Sensitivity Analysis of Data Transformation17**

**Supplementary Table 1**

The search strategy and results

| Database | Result | Search string |
| --- | --- | --- |
| Cochrane library | 164 | #1 ("college student" OR "university student" OR undergraduate* OR "undergraduate students" OR "universities students" OR undergraduate OR "college-student" OR college OR university):ti,ab,kw  #2 ("physical activity" OR "physical exercise" OR "sports activities" OR "sport movement" OR sport* OR "athletic sports" OR "aerobic exercise" OR "aerobic training" OR "resistance exercise" OR "muscle-strengthening exercise" OR "strength training" OR "fitness game" OR "circuit training" OR exercis* OR "physical endurance" OR "resistance training" OR aerobic OR "physical inactivity " OR "recreational activity" OR housework OR "non-exercise activity" OR "activities of daily" OR "physical fitness" OR bicycle OR walk OR run OR jogging OR swim OR "weight lifting" OR "motor activity" OR "leisure activities" OR recreation OR dancing OR gardening):ti,ab,kw  #3 (anxiety OR "anxiety disorder" OR "General Anxiety Disorder" OR GAD OR fear OR panic OR STAI OR "trait anxiety" OR "social anxiety"):ti,ab,kw  #4 ("psychological resilience" OR "psychological toughness" OR "Mental toughness" OR resilien* OR hardiness OR "social support" OR "social care"):ti,ab,kw  #5 #1 AND #2 AND #3 AND #4 |
| PsycINFO（via EBSCO） | 100 | #1 AB ("college student" OR "university student" OR undergraduate* OR "undergraduate students" OR "universities students" OR undergraduate OR "college-student" OR college OR university)  #2 AB ("physical activity" OR "physical exercise" OR "sports activities" OR "sport movement" OR sport* OR "athletic sports" OR "aerobic exercise" OR "aerobic training" OR "resistance exercise" OR "muscle-strengthening exercise" OR "strength training" OR "fitness game" OR "circuit training" OR exercis* OR "physical endurance" OR "resistance training" OR aerobic OR "physical inactivity " OR "recreational activity" OR housework OR "non-exercise activity" OR "activities of daily" OR "physical fitness" OR bicycle OR walk OR run OR jogging OR swim OR "weight lifting" OR "motor activity" OR "leisure activities" OR recreation OR dancing OR gardening)  #3 AB (anxiety OR "anxiety disorder" OR "General Anxiety Disorder" OR GAD OR fear OR panic OR STAI OR "trait anxiety" OR "social anxiety")  #4 AB ("psychological resilience" OR "psychological toughness" OR "Mental toughness" OR resilien* OR hardiness OR "social support" OR "social care")  #5 #1 AND #2 AND #3 AND #4 |
| PubMed | 143 | #1 "college student"[Title/Abstract] OR "university student"[Title/Abstract] OR undergraduate*[Title/Abstract] OR "undergraduate students"[Title/Abstract] OR "universities students"[Title/Abstract] OR undergraduate[Title/Abstract] OR "college-student"[Title/Abstract] OR college[Title/Abstract] OR university[Title/Abstract]  #2"physical activity"[Title/Abstract] OR "physical exercise"[Title/Abstract] OR "sports activities"[Title/Abstract] OR "sport movement"[Title/Abstract] OR sport*[Title/Abstract] OR "athletic sports"[Title/Abstract] OR "aerobic exercise"[Title/Abstract] OR "aerobic training"[Title/Abstract] OR "resistance exercise"[Title/Abstract] OR "muscle-strengthening exercise"[Title/Abstract] OR "strength training"[Title/Abstract] OR "fitness game"[Title/Abstract] OR "circuit training"[Title/Abstract] OR exercis*[Title/Abstract] OR "physical endurance"[Title/Abstract] OR "resistance training"[Title/Abstract] OR aerobic[Title/Abstract] OR "physical inactivity "[Title/Abstract] OR "recreational activity"[Title/Abstract] OR housework[Title/Abstract] OR "non-exercise activity"[Title/Abstract] OR "activities of daily"[Title/Abstract] OR "physical fitness"[Title/Abstract] OR bicycle[Title/Abstract] OR walk[Title/Abstract] OR run[Title/Abstract] OR jogging[Title/Abstract] OR swim[Title/Abstract] OR "weight lifting"[Title/Abstract] OR "motor activity"[Title/Abstract] OR "leisure activities"[Title/Abstract] OR recreation[Title/Abstract] OR dancing[Title/Abstract] OR gardening[Title/Abstract]  #3 anxiety[Title/Abstract] OR "anxiety disorder"[Title/Abstract] OR "General Anxiety Disorder"[Title/Abstract] OR GAD[Title/Abstract] OR fear[Title/Abstract] OR panic[Title/Abstract] OR STAI[Title/Abstract] OR "trait anxiety"[Title/Abstract] OR "social anxiety"[Title/Abstract]  #4 "psychological resilience"[Title/Abstract] OR "psychological toughness"[Title/Abstract] OR "Mental toughness"[Title/Abstract] OR resilien*[Title/Abstract] OR hardiness[Title/Abstract] OR "social support"[Title/Abstract] OR "social care"[Title/Abstract]  #5 #1 AND #2 AND #3 AND #4 |
| Scopus | 448 | #1 ( TITLE-ABS-KEY ( "college student" OR "university student" OR undergraduate* OR "undergraduate students" OR "universities students" OR undergraduate OR "college-student" OR college OR university ) )  #2 ( TITLE-ABS-KEY ( "physical activity" OR "physical exercise" OR "sports activities" OR "sport movement" OR sport* OR "athletic sports" OR "aerobic exercise" OR "aerobic training" OR "resistance exercise" OR "muscle-strengthening exercise" OR "strength training" OR "fitness game" OR "circuit training" OR exercis* OR "physical endurance" OR "resistance training" OR aerobic OR "physical inactivity " OR "recreational activity" OR housework OR "non-exercise activity" OR "activities of daily" OR "physical fitness" OR bicycle OR walk OR run OR jogging OR swim OR "weight lifting" OR "motor activity" OR "leisure activities" OR recreation OR dancing OR gardening ) )  #3 ( TITLE-ABS-KEY ( anxiety OR "anxiety disorder" OR "General Anxiety Disorder" OR GAD OR fear OR panic OR STAI OR "trait anxiety" OR "social anxiety" ) )  #4 ( TITLE-ABS-KEY ( "psychological resilience" OR "psychological toughness" OR "Mental toughness" OR resilien* OR hardiness OR "social support" OR "social care" ) )  #5 #1 AND #2 AND #3 AND #4 |
| Web of Science | 672 | #1 TS=("college student" OR "university student" OR undergraduate* OR "undergraduate students" OR "universities students" OR undergraduate OR "college-student" OR college OR university)  #2 TS=("physical activity" OR "physical exercise" OR "sports activities" OR "sport movement" OR sport* OR "athletic sports" OR "aerobic exercise" OR "aerobic training" OR "resistance exercise" OR "muscle-strengthening exercise" OR "strength training" OR "fitness game" OR "circuit training" OR exercis* OR "physical endurance" OR "resistance training" OR aerobic OR "physical inactivity " OR "recreational activity" OR housework OR "non-exercise activity" OR "activities of daily" OR "physical fitness" OR bicycle OR walk OR run OR jogging OR swim OR "weight lifting" OR "motor activity" OR "leisure activities" OR recreation OR dancing OR gardening)  #3 TS=(anxiety OR "anxiety disorder" OR "General Anxiety Disorder" OR GAD OR fear OR panic OR STAI OR "trait anxiety" OR "social anxiety")  #4 TS=("psychological resilience" OR "psychological toughness" OR "Mental toughness" OR resilien* OR hardiness OR "social support" OR "social care")  #5 #1 AND #2 AND #3 AND #4 |
| CNKI | 577 | #1 SU=（大学生+本科生+学院+大学+高校）  #2 SU=（运动+体育活动+身体活动+体育锻炼+体力活动+有氧训练+阻力运动+力量训练+健身+电路训练+锻炼+身体耐力+阻力训练+有氧+娱乐活动+家务+日常活动+健身+自行车+步行+跑步+慢跑+游泳+举重+休闲活动+娱乐+跳舞+园艺）  #3 SU=（社会支持+感知社会支持+社会关怀+心理韧性+心理弹性+复原力+坚韧性）  #4 SU=（焦虑+焦虑症+焦虑障碍+社交焦虑+广泛性焦虑障碍+特质焦虑）  #5 #1 AND #2 AND #3 AND #4 |
| Wanfang | 166 | #1主题:(大学生 OR 本科生 OR 学院 OR 大学 OR 高校)  #2主题:(运动 OR 体育活动 OR 身体活动 OR 体育锻炼 OR 体力活动)  #3主题:(焦虑 OR 焦虑症 OR 焦虑障碍 OR 社交焦虑 OR 广泛性焦虑障碍 OR 特质焦虑)  #4主题:(社会支持 OR 感知社会支持 OR 社会关怀 OR 心理韧性 OR 心理弹性 OR 复原力 OR 坚韧性)  #5 #1 AND #2 AND #3 AND #4 |
| VIP | 73 | #1 R=(大学生 OR 本科生 OR 学院 OR 大学 OR 高校)  #2 R=(运动 OR 体育活动 OR 身体活动 OR 体育锻炼 OR 体力活动 OR 有氧训练 OR 阻力运动 OR 力量训练 OR 健身 OR 电路训练 OR 锻炼 OR 身体耐力 OR 阻力训练 OR 有氧 OR 娱乐活动 OR 家务 OR 日常活动 OR 自行车 OR 步行 OR 跑步 OR 慢跑 OR 游泳 OR 举重 OR 休闲活动 OR 娱乐 OR 跳舞 OR 园艺)  #3 R=(焦虑 OR 焦虑症 OR 焦虑障碍 OR 社交焦虑 OR 广泛性焦虑障碍 OR 特质焦虑)  #4 R=(社会支持 OR 感知社会支持 OR 社会关怀 OR 心理韧性 OR 心理弹性 OR 复原力 OR 坚韧性)  #5 #1 AND #2 AND #3 AND #4 |

**Supplementary Table 2**

Other characteristics of studies included in the meta-analysis

| Study name | Physical Activity Measurement Tools | Psychological Resilience Measurement Tools | Social Support Measurement Tools | Anxiety Measurement Tools | Pairwise Relationships | Pearson correlation coefficient | Spearman correlation coefficient | Physical Activity Scale α | Psychological Resilience Scale α | Social Support Scale α | Anxiety Scale α |
| --- | --- | --- | --- | --- | --- | --- | --- | --- | --- | --- | --- |
| Lian.（2022） | PARS-3 | APRS | PSSS | Nr | PA-PR | 0.1 |  | Nr | 0.75 | 0.88 | Nr |
|  |  |  |  |  | PA-SS | 0.109 |  |  |  |  |  |
|  |  |  |  |  | PR-SS | 0.474 |  |  |  |  |  |
| Cui.(2024) | PARS-3 | CD-RISC | SSRS | Nr | PA-PR | 0.42 |  | 0.816 | 0.96 | 0.921 | Nr |
|  |  |  |  |  | PA-SS | 0.45 |  |  |  |  |  |
|  |  |  |  |  | PR-SS | 0.59 |  |  |  |  |  |
| Guo.(2023) | PARS-3 | APRS | Nr | IAS | PA-PR | 0.656 | 0.639 | 0.888 | 0.957 | Nr | 0.924 |
|  |  |  |  |  | PA-ANX | -0.548 | -0.53 |  |  |  |  |
|  |  |  |  |  | PR-ANX | -0.691 | -0.673 |  |  |  |  |
| Cui et al.(2023) | PARS-3 | CD-RISC | Nr | SAS-CR | PA-PR | 0.245 |  | 0.736 | 0.944 | Nr | 0.879 |
|  |  |  |  |  | PA-ANX | -0.186 |  |  |  |  |  |
|  |  |  |  |  | PR-ANX | -0.341 |  |  |  |  |  |
| Liang.(2023) | PARS-3 | Nr | SSRS | IAS | PA-SS | 0.076 |  | 0.856 | Nr | 0.88 | 0.936 |
|  |  |  |  |  | PA-ANX | -0.076 |  |  |  |  |  |
|  |  |  |  |  | SS-ANX | -0.401 |  |  |  |  |  |
| Liu.(2020) | PARS-3 | APRS | Nr | DASS-21 | PA-PR | 0.253 |  | Nr | 0.75 | Nr | 0.881 |
|  |  |  |  |  | PA-ANX | -0.34 |  |  |  |  |  |
|  |  |  |  |  | PR-ANX | -0.322 |  |  |  |  |  |
| Liao et al.(2025) | PARS-3 | CD-RISC | Nr | SAS | PA-PR | 0.21 |  | 0.6 | 0.95 | Nr | 0.86 |
|  |  |  |  |  | PA-ANX | -0.11 |  |  |  |  |  |
|  |  |  |  |  | PR-ANX | -0.23 |  |  |  |  |  |
| Wei.(2024) | PARS-3 | CD-RISC | Nr | SAS | PA-PR | 0.481 |  | 0.732 | 0.917 | Nr | 0.828 |
|  |  |  |  |  | PA-ANX | -0.487 |  |  |  |  |  |
|  |  |  |  |  | PR-ANX | -0.482 |  |  |  |  |  |
| Li Jinying.(2023) | PARS-3 | APRS | SSA | Nr | PA-PR | 0.096 |  | 0.903 | 0.855 | 0.933 | Nr |
|  |  |  |  |  | PA-SS | 0.067 |  |  |  |  |  |
|  |  |  |  |  | PR-SS | 0.306 |  |  |  |  |  |
| Ding et al.(2025) | PARS-3 | APRS | PSSS | Nr | PA-PR | 0.12 |  | 0.73 | 0.9 | 0.96 | Nr |
|  |  |  |  |  | PA-SS | 0.19 |  |  |  |  |  |
|  |  |  |  |  | PR-SS | 0.38 |  |  |  |  |  |
| Li Pengcheng.(2023) | PARS-3 | CD-RISC | Nr | DASS-21 | PA-PR | 0.337 |  | 0.901 | 0.864 | Nr | 0.913 |
|  |  |  |  |  | PA-ANX | -0.517 |  |  |  |  |  |
|  |  |  |  |  | PR-ANX | -0.587 |  |  |  |  |  |
| Wang Zhifeng et al.(2024) | PARS-3 | CD-RISC | Nr | SCL-90 | PA-PR | 0.61 |  | Nr | 0.93 | Nr | 0.89 |
|  |  |  |  |  | PA-ANX | -0.67 |  |  |  |  |  |
|  |  |  |  |  | PR-ANX | -0.48 |  |  |  |  |  |
| Zhu and Liu.(2021) | PARS-3 | CD-RISC | Nr | IAS | PA-PR | 0.551 |  | 0.614 | 0.963 | Nr | 0.815 |
|  |  |  |  |  | PA-ANX | -0.42 |  |  |  |  |  |
|  |  |  |  |  | PR-ANX | -0.461 |  |  |  |  |  |
| Xiu.(2021) | PARS-3 | APRS | Nr | DASS-21 | PA-PR | 0.925 |  | Nr | 0.85 | Nr | Nr |
|  |  |  |  |  | PA-ANX | -0.907 |  |  |  |  |  |
|  |  |  |  |  | PR-ANX | -0.925 |  |  |  |  |  |
| Demi̇r and Barut.（2020） | IPAQ | SPRS | Nr | BAI | PR-ANX | -0.255 |  | Nr | 0.86 | Nr | 0.93 |
| Deng and Wang.（2024） | PARS-3 | Nr | SSS | SAS | PA-SS | 0.354 |  | 0.896 | Nr | 0.838 | 0.947 |
|  |  |  |  |  | PA-ANX | -0.326 |  |  |  |  |  |
|  |  |  |  |  | SS-ANX | -0.382 |  |  |  |  |  |
| Jiang et al.（2025） | PARS-3 | CD-RISC | Nr | IAS | PA-PR | 0.138 |  | 0.879 | 0.956 | Nr | 0.96 |
|  |  |  |  |  | PA-ANX | -0.038 |  |  |  |  |  |
|  |  |  |  |  | PR-ANX | -0.607 |  |  |  |  |  |
| Jiang and Wang.（2025） | PARS-3 | APRS | Nr | SPAS | PA-PR | 0.382 |  | 0.85 | 0.94 | Nr | 0.83 |
|  |  |  |  |  | PA-ANX | -0.49 |  |  |  |  |  |
|  |  |  |  |  | PR-ANX | -0.284 |  |  |  |  |  |
| Johannes et al.（2024） | IPAQ-SF | Nr | PSSS | DASS-21 | SS-ANX | -0.3 |  | Nr | Nr | 0.875 | 0.856 |
| Liu,S. et al.（2023） | PARS-3 | CD-RISC | Nr | SAS | PA-PR | 0.191 |  | Nr | 0.9 | Nr | Nr |
|  |  |  |  |  | PA-ANX | -0.061 |  |  |  |  |  |
|  |  |  |  |  | PR-ANX | -0.403 |  |  |  |  |  |
| Liu and Shi.（2023） | PARS-3 | Nr | SSRS | GAD-7 | PA-SS | 0.225 |  | 0.758 | Nr | Nr | Nr |
|  |  |  |  |  | PA-ANX | -0.539 |  |  |  |  |  |
|  |  |  |  |  | SS-ANX | -0.269 |  |  |  |  |  |
| Liu et al.（2024） | PARS-3 | CD-RISC | Nr | DASS-21 | PA-PR | 0.27 |  | Nr | 0.87 | Nr | Nr |
|  |  |  |  |  | PA-ANX | -0.094 |  |  |  |  |  |
|  |  |  |  |  | PR-ANX | -0.186 |  |  |  |  |  |
| Maier and James.（2014） | HNEQ | Nr | ISEL | NEO-PI | PA-SS | 0.103 |  | Nr | Nr | 0.83 | 0.82 |
|  |  |  |  |  | PA-ANX | -0.182 |  |  |  |  |  |
|  |  |  |  |  | SS-ANX | -0.21 |  |  |  |  |  |
| Margraf et al.（2023） | Nr | RS-11 | F-SozU K-14 | DASS-21 | PR-SS | 0.309 |  | Nr | 0.904 | 0.938 | 0.795 |
|  |  |  |  |  | PR-ANX | -0.221 |  |  |  |  |  |
|  |  |  |  |  | SS-ANX | -0.333 |  |  |  |  |  |
|  | Nr | RS-11 | F-SozU K-14 | DASS-21 | PR-SS | 0.299 |  | Nr | 0.793 | 0.948 | 0.737 |
|  |  |  |  |  | PR-ANX | -0.322 |  |  |  |  |  |
|  |  |  |  |  | SS-ANX | -0.151 |  |  |  |  |  |
| Qin et al.(2024) | IPAQ | CD-RISC | PSSS | GAD-7 | PA-PR | 0.29 | 0.278 | Nr | 0.97 | 0.952 | 0.945 |
|  |  |  |  |  | PA-SS | 0.371 | 0.356 |  |  |  |  |
|  |  |  |  |  | PA-ANX | 0.312 | -0.299 |  |  |  |  |
|  |  |  |  |  | PR-SS | 0.514 | 0.496 |  |  |  |  |
|  |  |  |  |  | PR-ANX | -0.38 | -0.365 |  |  |  |  |
|  |  |  |  |  | SS-ANX | -0.368 | -0.353 |  |  |  |  |
| Wu et al.(2024) | IPAQ-SF | CD-RISC | Nr | IAS | PA-PR | 0.3 |  | 0.87 | 0.93 | Nr | 0.89 |
|  |  |  |  |  | PA-ANX | -0.23 |  |  |  |  |  |
|  |  |  |  |  | PR-ANX | -0.31 |  |  |  |  |  |
| Yue et al.(2022) | PARS-3 | CD-RISC | Nr | DASS-21 | PA-PR | 0.201 | 0.192 | Nr | 0.748 | Nr | 0.882 |
| Zhao,H, et al.(2025) | PARS-3 | MTS-CS | Nr | SAS | PA-PR | 0.162 |  | 0.668 | 0.889 | Nr | 0.801 |
|  |  |  |  |  | PA-ANX | -0.326 |  |  |  |  |  |
|  |  |  |  |  | PR-ANX | -0.355 |  |  |  |  |  |
| Zheng et al.(2024) | PARS-3 | CD-RISC | SSRS | SAS | PA-PR | 0.69 |  | 0.71 | 0.95 | 0.76 | 0.86 |
|  |  |  |  |  | PA-SS | 0.48 |  |  |  |  |  |
|  |  |  |  |  | PA-ANX | -0.73 |  |  |  |  |  |
|  |  |  |  |  | PR-SS | 0.44 |  |  |  |  |  |
|  |  |  |  |  | PR-ANX | -0.62 |  |  |  |  |  |
|  |  |  |  |  | SS-ANX | -0.48 |  |  |  |  |  |

Abbreviation: Nr, not reported

PA：IPAQ，International Physical Activity Questionnaire；PARS-3， Physical Activity Rating Scale；PEQ，Physical Exercise Questionnaire；HNEQ，Houston Non-Exercise Questionnaire

PR：CD-RISC，ConnorDavidson Resilience Scale；APRS，Adolescent Psychological Resilience Scale；SPRS，Short Psychological Resilience Scale；RS-14，11-item short version of the Wagnild and Young Resilience Scale；MTS-CS，Mental Toughness Scale for College Students

SS：PSSS，Perceived Social Support Scale；SSRS，Social Support Rating Scale；SSA，Social Support Appraisals；SSS，social support scale；ISEL，Interpersonal Support Evaluation List-12；F-SozU K-14，14-item Questionnaire- Social Support14

ANX：GAD -7，Generalized Anxiety Disorder-7；IAS，Interaction Anxiousness Scale；SAS，Self-Rating Anxiety Scale；DASS-21，Depression Anxiety Stress Scales - 21 Items；SCL-90，Symptom Checklist-90；BAI，Beck Anxiety Inventory；SPAS，Social Physique Anxiety Scale；SIAS-6，Social Interaction Anxiety Scale；NEO-PI，NEO Personality Inventory

**Supplementary Table 3**

Quality Assessment for each observational study individually

| **Study** | *Prospective study* | *Probability sampling* | *Sample size justification* | *Multi-site sampling* | *Protection of anonymity* | *Response rate ≥ 60%* | *Reliable independent variable measurement* | *Valid instrument used for independent variable measurement* | *Valid instrument used for dependent variable measurement* | *Internal consistency ≥ 0.70 for dependent variable scale (if used)* | *Use of a theoretical framework* | *Analysis of correlations for multiple outcomes* | *Management of outliers* | ***Overall Score*** | ***Classification of quality*** |
| --- | --- | --- | --- | --- | --- | --- | --- | --- | --- | --- | --- | --- | --- | --- | --- |
| Demi̇r and Barut.（2020） | 0 | 1 | 1 | 0 | 0 | 0 | 1 | 1 | 1 | 2 | 0 | 1 | 0 | 8 | Medium |
| Deng and Wang.（2024） | 0 | 0 | 1 | 1 | 1 | 1 | 1 | 1 | 1 | 2 | 1 | 1 | 1 | 12 | High |
| Jiang et al.（2025） | 0 | 0 | 1 | 1 | 1 | 1 | 1 | 1 | 1 | 2 | 1 | 1 | 1 | 12 | High |
| Jiang and Wang.（2025） | 0 | 0 | 1 | 1 | 0 | 1 | 1 | 1 | 1 | 2 | 1 | 1 | 1 | 11 | High |
| Johannes et al.（2024） | 0 | 0 | 1 | 0 | 1 | 0 | 1 | 1 | 1 | 2 | 0 | 1 | 0 | 8 | Medium |
| Liu,S. et al.（2023） | 0 | 1 | 1 | 1 | 1 | 1 | 1 | 1 | 1 | 2 | 0 | 1 | 1 | 12 | High |
| Liu and Shi.（2023） | 0 | 0 | 1 | 0 | 1 | 1 | 1 | 1 | 1 | 2 | 0 | 1 | 1 | 10 | High |
| Liu et al.（2024） | 0 | 1 | 1 | 1 | 1 | 1 | 1 | 1 | 1 | 2 | 1 | 1 | 1 | 13 | High |
| Maier and James.（2014） | 0 | 0 | 1 | 0 | 0 | 0 | 1 | 1 | 1 | 0 | 1 | 1 | 0 | 6 | Medium |
| Qin et al.(2024) | 0 | 0 | 1 | 0 | 1 | 1 | 1 | 1 | 1 | 2 | 1 | 1 | 1 | 11 | High |
| Wu et al.(2024) | 0 | 0 | 1 | 1 | 0 | 0 | 1 | 1 | 1 | 2 | 1 | 1 | 1 | 10 | High |
| Yue et al.(2022) | 0 | 0 | 1 | 1 | 0 | 1 | 1 | 1 | 1 | 2 | 0 | 1 | 0 | 9 | Medium |
| Zhao,H, et al.(2025) | 0 | 1 | 1 | 1 | 0 | 1 | 1 | 1 | 1 | 2 | 1 | 1 | 1 | 12 | High |
| Zheng et al.(2024) | 0 | 0 | 1 | 1 | 0 | 1 | 1 | 1 | 1 | 2 | 1 | 1 | 0 | 10 | High |
| Lian.（2022） | 0 | 0 | 1 | 1 | 1 | 0 | 1 | 1 | 1 | 2 | 1 | 1 | 1 | 12 | High |
| Cui.(2024) | 0 | 0 | 1 | 1 | 0 | 1 | 1 | 1 | 1 | 2 | 1 | 1 | 1 | 11 | High |
| Guo.(2023) | 0 | 0 | 1 | 1 | 1 | 1 | 1 | 1 | 1 | 2 | 1 | 1 | 1 | 12 | High |
| Cui et al.(2023) | 0 | 0 | 1 | 0 | 0 | 1 | 1 | 1 | 1 | 2 | 1 | 1 | 1 | 10 | High |
| Liang.(2023) | 0 | 0 | 1 | 1 | 0 | 1 | 1 | 1 | 1 | 2 | 1 | 1 | 0 | 10 | High |
| Liu.(2020) | 0 | 1 | 1 | 1 | 0 | 1 | 1 | 1 | 1 | 2 | 1 | 1 | 1 | 12 | High |
| Liao et al.(2025) | 0 | 0 | 1 | 0 | 0 | 1 | 1 | 1 | 1 | 2 | 1 | 1 | 1 | 10 | High |
| Wei.(2024) | 0 | 0 | 1 | 0 | 0 | 0 | 1 | 1 | 1 | 2 | 1 | 1 | 0 | 8 | Medium |
| Li Jinying.(2023) | 0 | 0 | 1 | 1 | 0 | 1 | 1 | 1 | 1 | 2 | 1 | 1 | 1 | 11 | High |
| Ding et al.(2025) | 0 | 0 | 1 | 1 | 0 | 1 | 1 | 1 | 1 | 2 | 1 | 1 | 1 | 11 | High |
| Li Pengcheng.(2023) | 0 | 0 | 1 | 0 | 1 | 1 | 1 | 1 | 1 | 2 | 1 | 1 | 1 | 11 | High |
| Wang Zhifeng et al.(2024) | 0 | 0 | 1 | 1 | 0 | 1 | 1 | 1 | 1 | 2 | 1 | 1 | 1 | 11 | High |
| Zhu and Liu.(2021) | 0 | 0 | 1 | 0 | 0 | 1 | 1 | 1 | 1 | 2 | 1 | 1 | 1 | 10 | High |

**Supplementary Table 4**

Quality Assessment for RCT study

| Bias | Authors’ judgment | Support for judgment |
| --- | --- | --- |
| Random sequence generation (selection bias) | Low risk | The study subjects were university students with weak physical constitutions, randomly divided into 3 classes for physical education. |
| Allocation concealment (selection bias) | High risk | Allocation concealment measures were not reported. |
| Blinding of participants and researchers (performance bias) | Low risk | A single-blind experiment was used where students were unaware of the details. |
| Blinding of outcome assessment (detection bias) | Unclear risk | It was not specified whether blinding measures were taken for outcome assessment, and the scales were mostly self-rated. |
| Incomplete outcome data (attrition bias) | Low risk | It was reported that 3 questionnaires were invalid and excluded, with an actual inclusion of 72 participants and no other significant sample attrition. |
| Selective reporting (reporting bias) | Low risk | All pre-specified outcomes were reported. |
| Other bias | Unclear risk | Some analyses were only for voluntary participants and the physically weak group, indicating a possibility of selection/volunteer bias, but no serious anomalies were observed. |

**Supplementary Table 5**

Quality Assessment for Prospective cohort study study

| Domain | Project | Score |
| --- | --- | --- |
| Selection | Representativeness of the exposed cohort | 0 |
|  | Representativeness of the non-exposed cohort | 1 |
|  | Determination of Exposure Factors | 0 |
|  | It is certain that at the beginning of the study, there were no outcome indicators to be observed. | 1 |
| Comparability | Consider the comparability between the exposed and unexposed groups during design and statistical analysis | 2 |
| Outcome | Assessment of outcome | 0 |
|  | Was follow up long enough for outcomes to occur | 1 |
|  | Completeness of the exposed and non-exposed groups | 0 |
| Overall Score |  | 5 |

**Supplementary Table 6**

Measurement error and factor loading(TSSEM-Stage 2)

|  | PA | PR | SS | ANX |
| --- | --- | --- | --- | --- |
| α | 0.788647059 | 0.892 | 0.892916667 | 0.871772727 |
| Measurement error（1-α） | 0.211352941 | 0.108 | 0.107083333 | 0.128227273 |
| Factor loading（square root of α） | 0.888058027 | 0.944457516 | 0.944942679 | 0.933687703 |

α：Average Cronbach's coefficient of the variables

**Supplementary Table 7**

Sensitivity Analysis of Data Transformation

| Pairwise Relationships | Effect Size(r) | | Heterogeneity（I^2^） | | P | |
| --- | --- | --- | --- | --- | --- | --- |
|  | Group 1 | Group 2 | Group 1 | Group 2 | Group 1 | Group 2 |
| PA-ANX | -0.368 | -0.389 | 98% | 99% | 0.000 | 0.000 |
| PA-PR | 0.375 | 0.37 | 98% | 98% | 0.000 | 0.000 |
| PA-SS | 0.247 | 0.232 | 95% | 96% | 0.000 | 0.000 |
| PR-ANX | -0.443 | -0.426 | 97% | 97% | 0.000 | 0.000 |
| PR-SS | 0.418 | 0.404 | 96% | 96% | 0.000 | 0.000 |
| SS-ANX | -0.322 | -0.316 | 95% | 95% | 0.000 | 0.000 |

Group 1：Mixed dataset with Spearman-transformed data；Group 2：Studies reporting only Pearson correlation coefficients

**
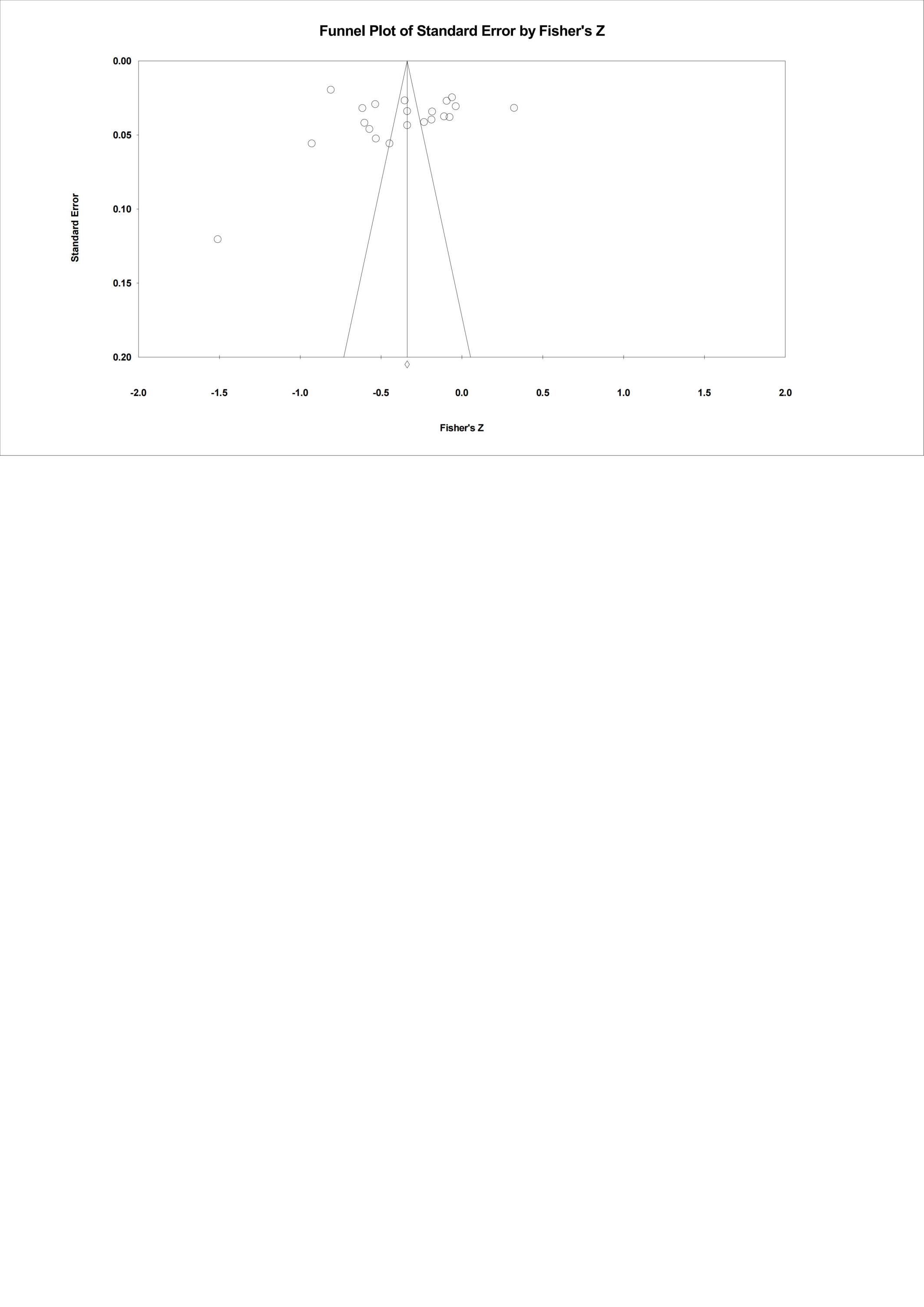
**

**Supplementary Figure 1.**Funnel plot of the correlation between physical activity and anxiety

**
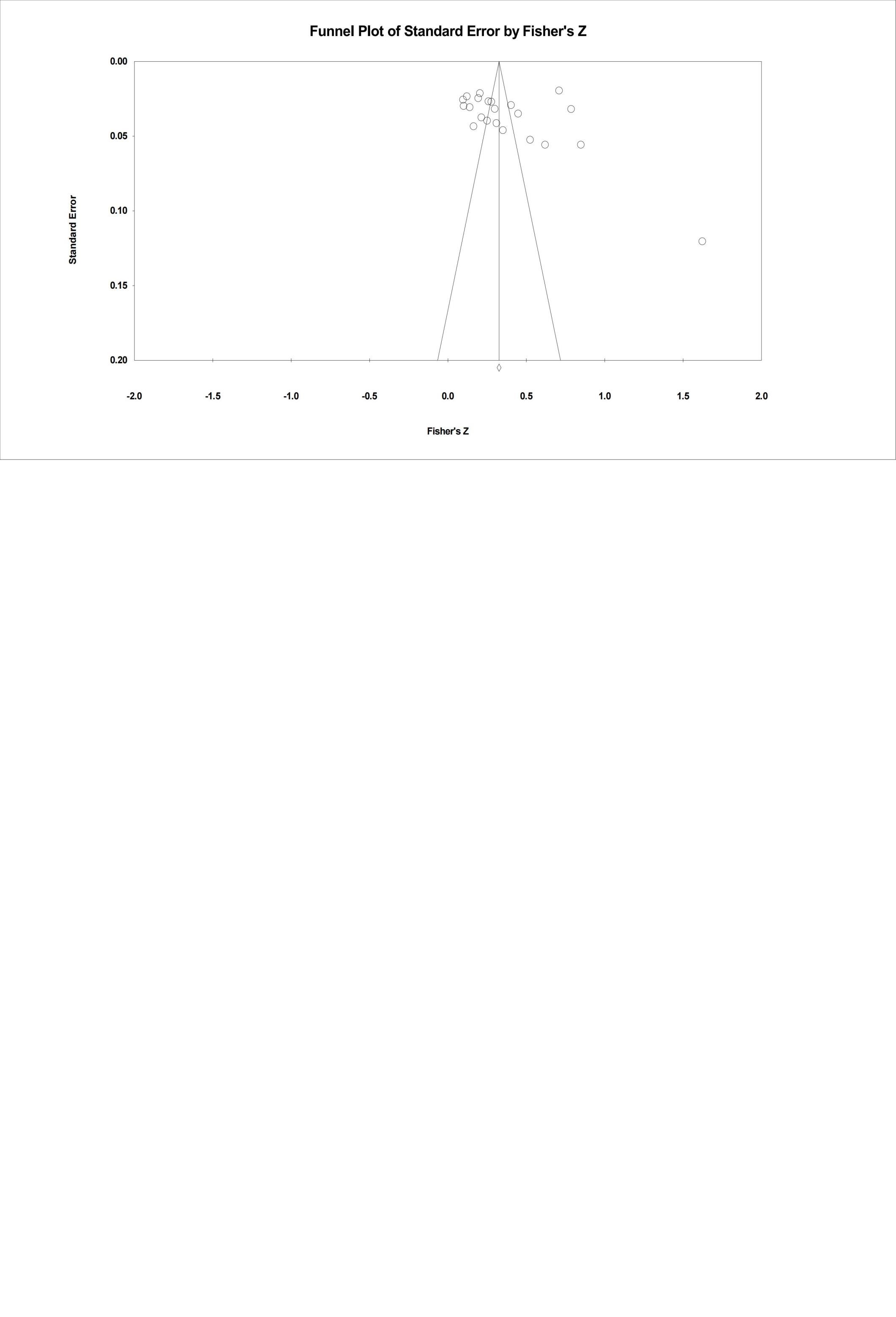
**

**Supplementary Figure 2.**Funnel plot of the correlation between physical activity and psychological resilience

**
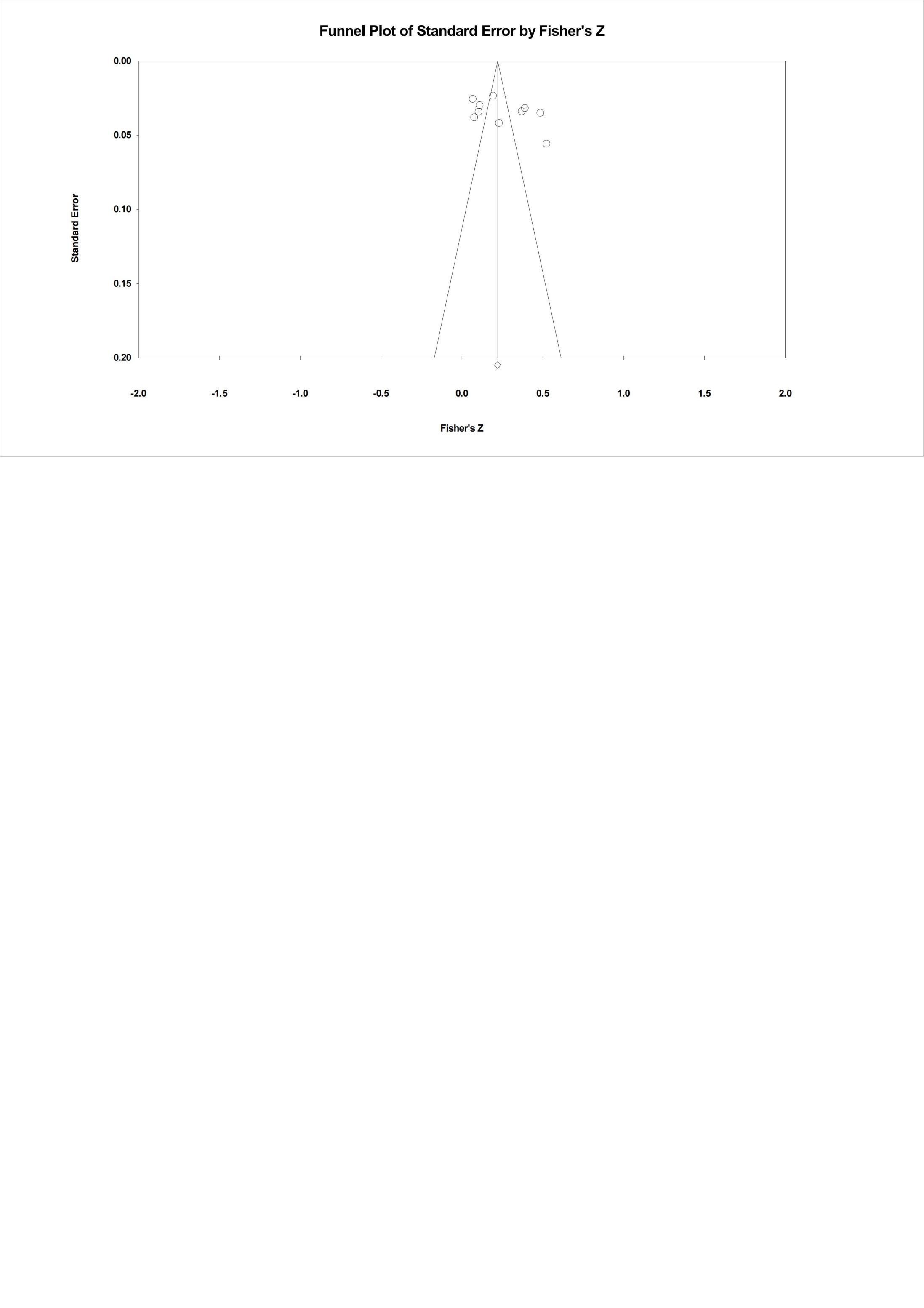
**

**Supplementary Figure 3.**Funnel plot of the correlation between physical activity and social support

**
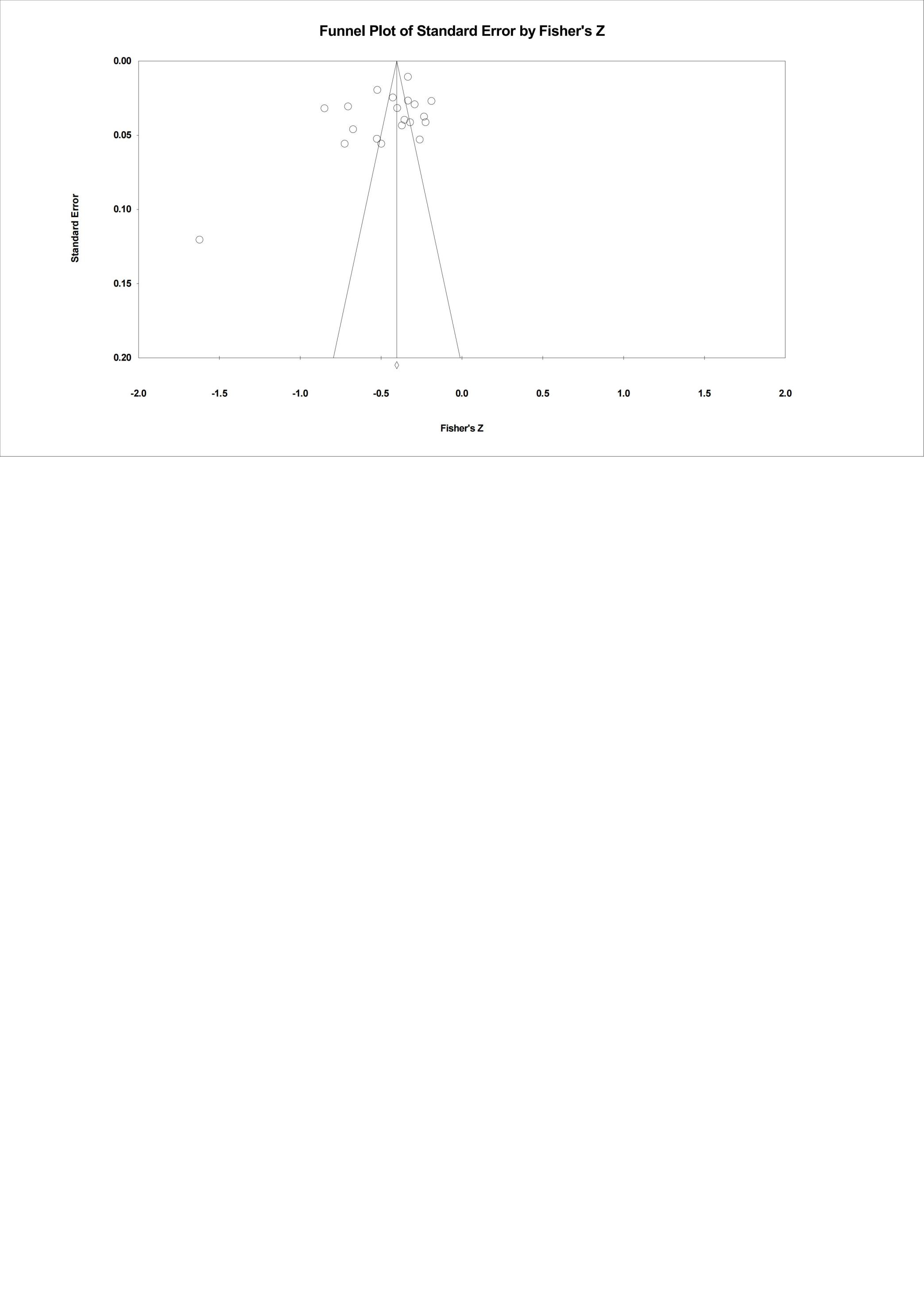
**

**Supplementary Figure 4.**Funnel plot of the correlation between psychological resilience and anxiety

**
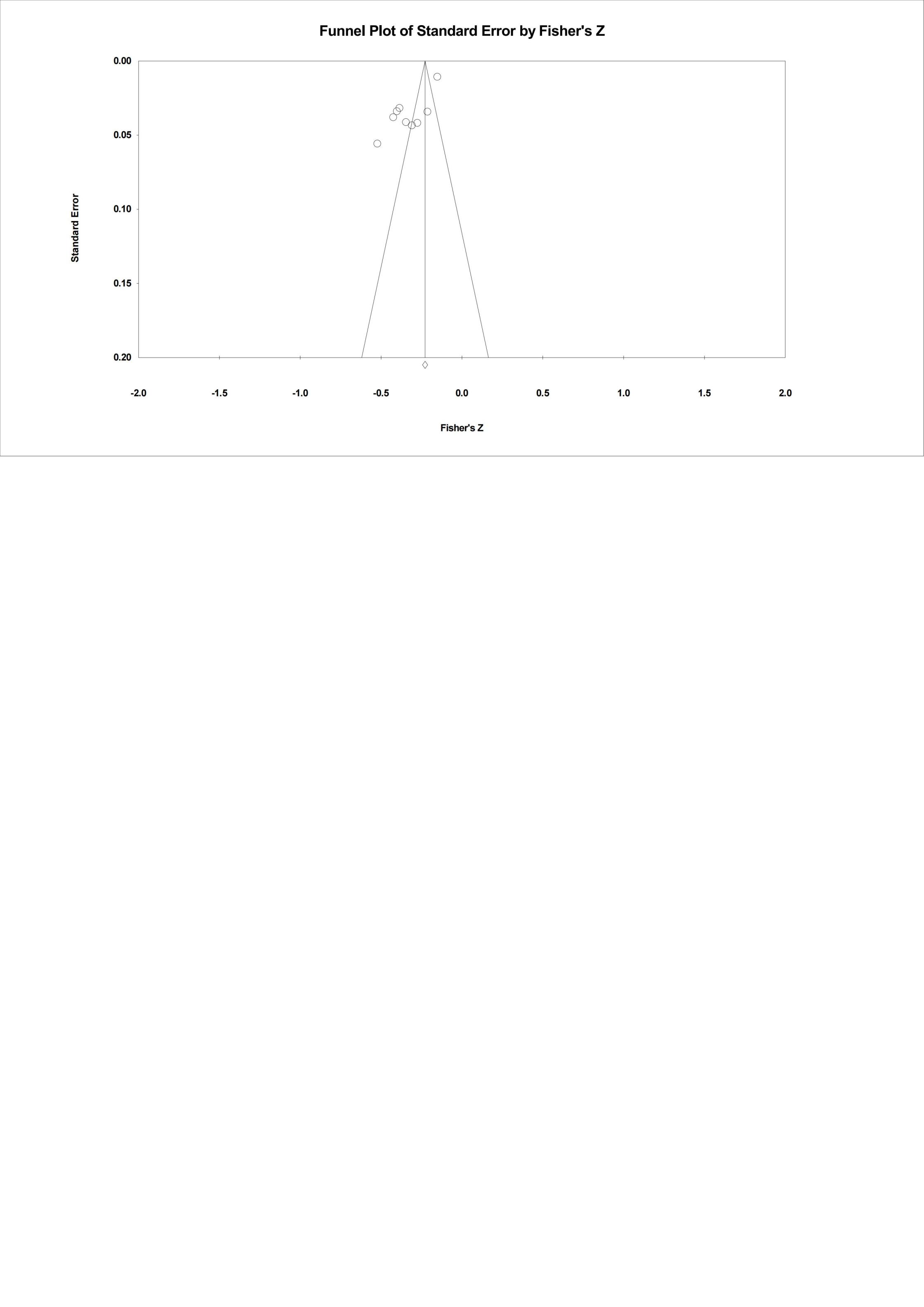
**

**Supplementary Figure 5.**Funnel plot of the correlation between social support and anxiety

**
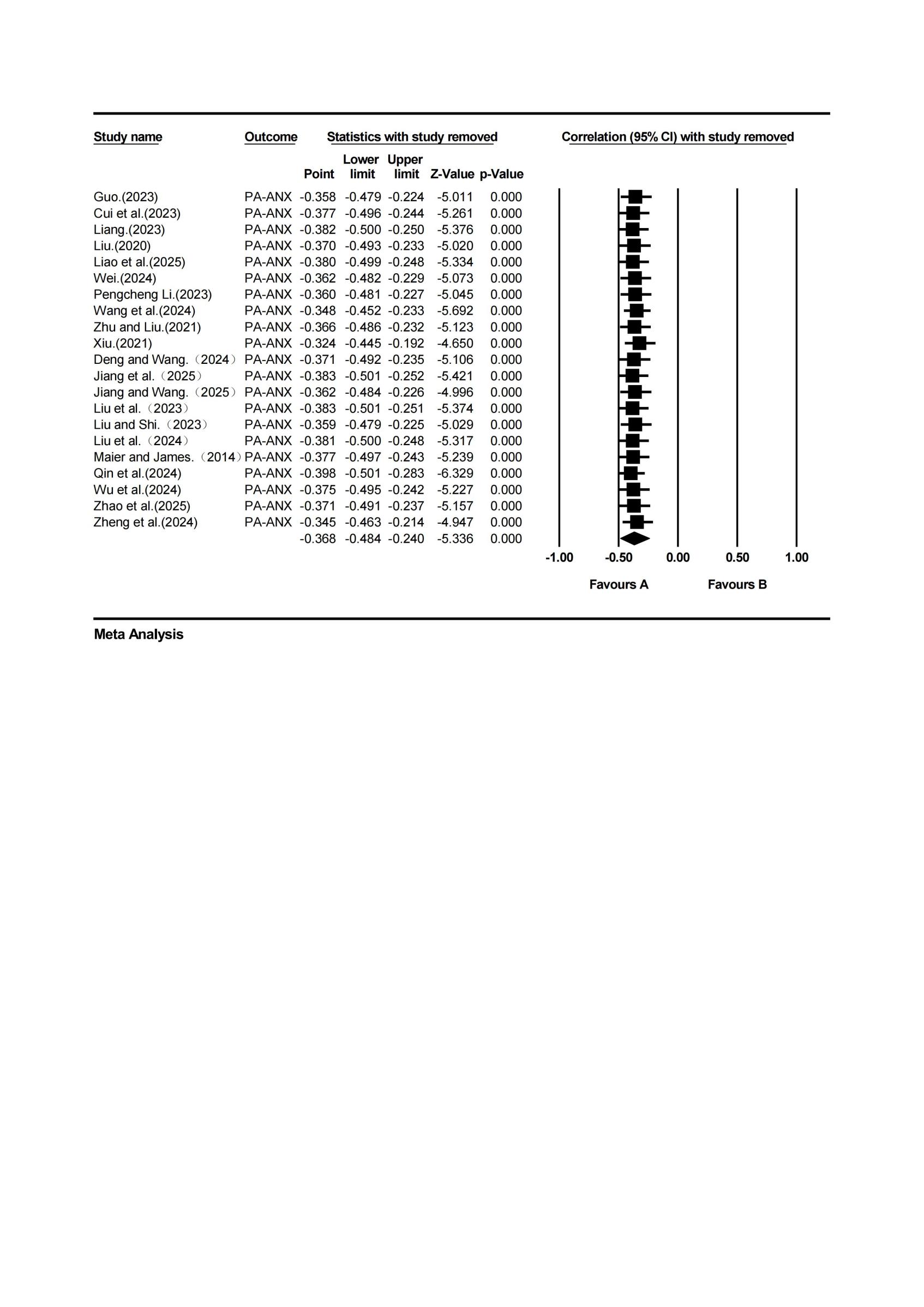
**

**Supplementary Figure 6.**Sensitivity analysis of the correlation between physical activity and anxiety


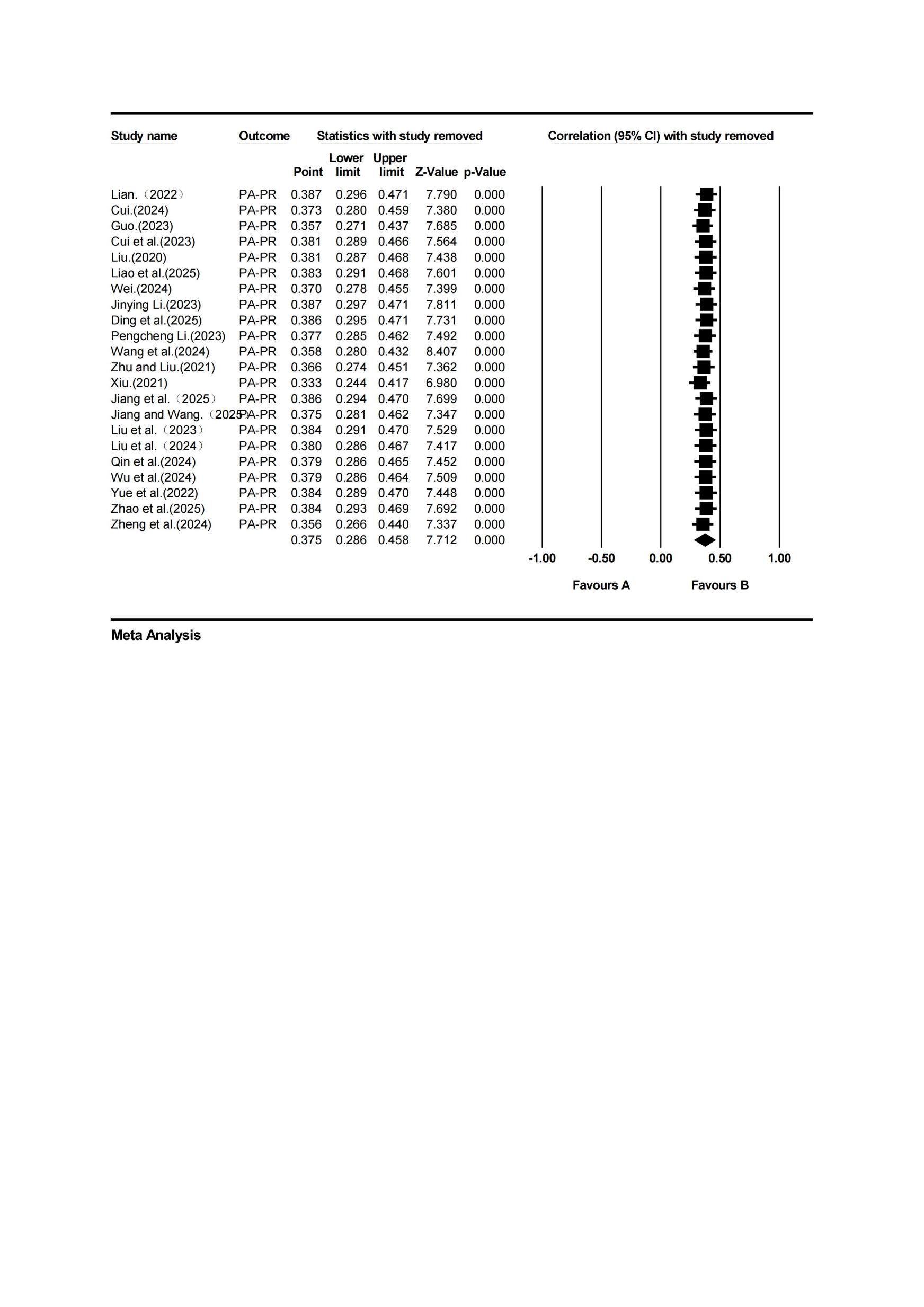


**Supplementary Figure 7.**Sensitivity analysis of the correlation between physical activity and psychological resilience


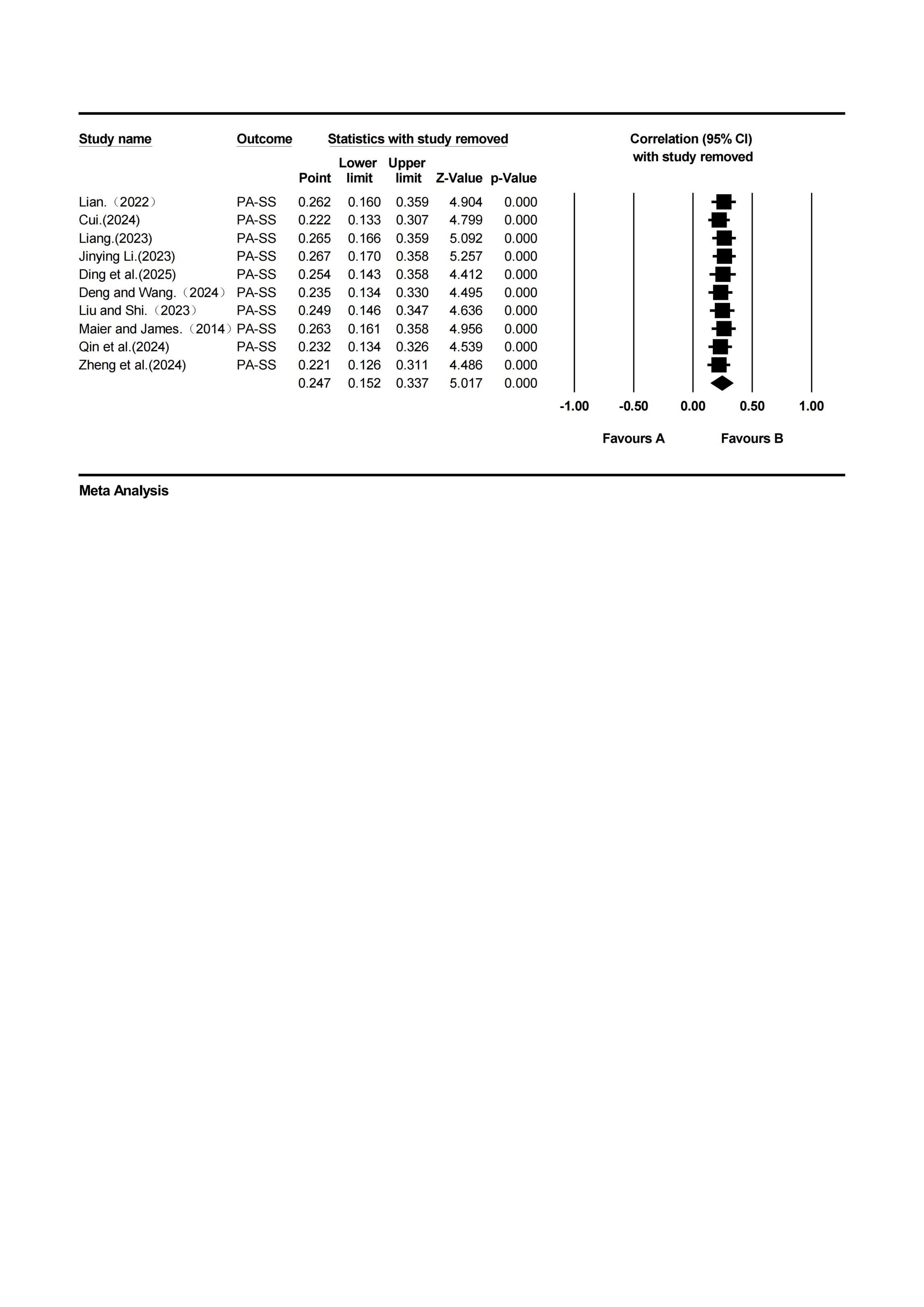


**Supplementary Figure 8.**Sensitivity analysis of the correlation between physical activity and social support


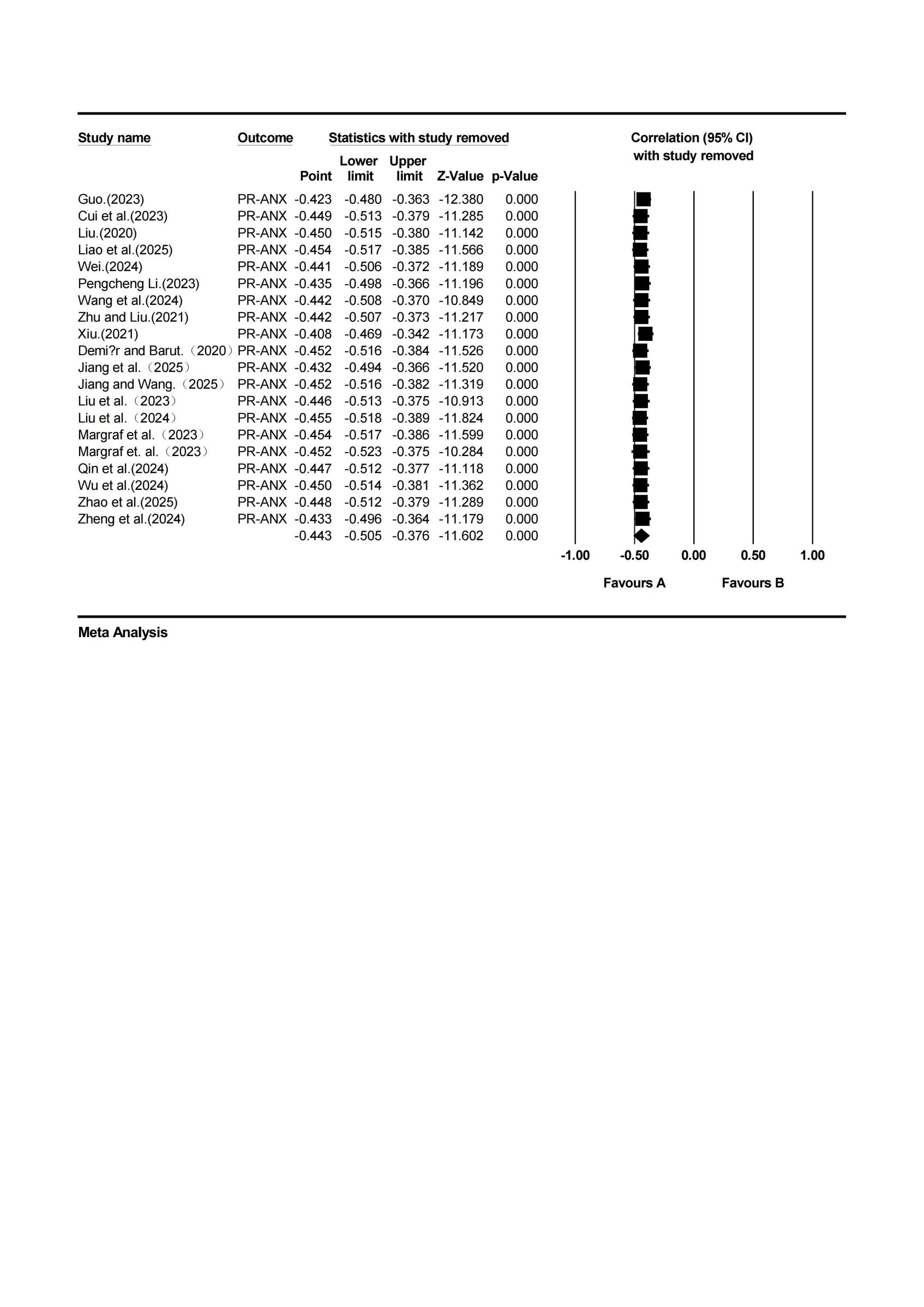


**Supplementary Figure 9.**Sensitivity analysis of the correlation between psychological resilience and anxiety

**
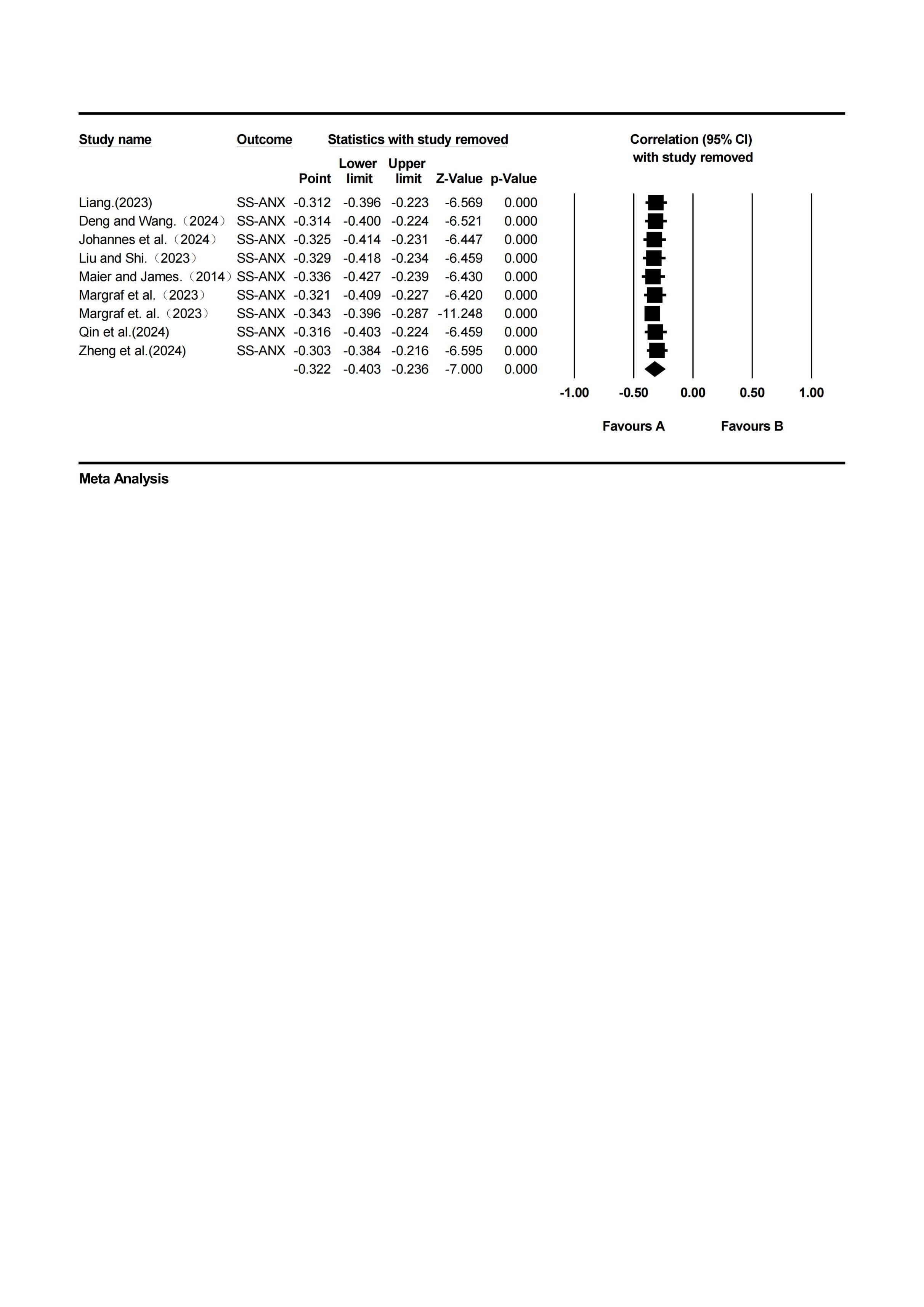
**

**Supplementary Figure 10.**Sensitivity analysis of the correlation between social support and anxiety

**
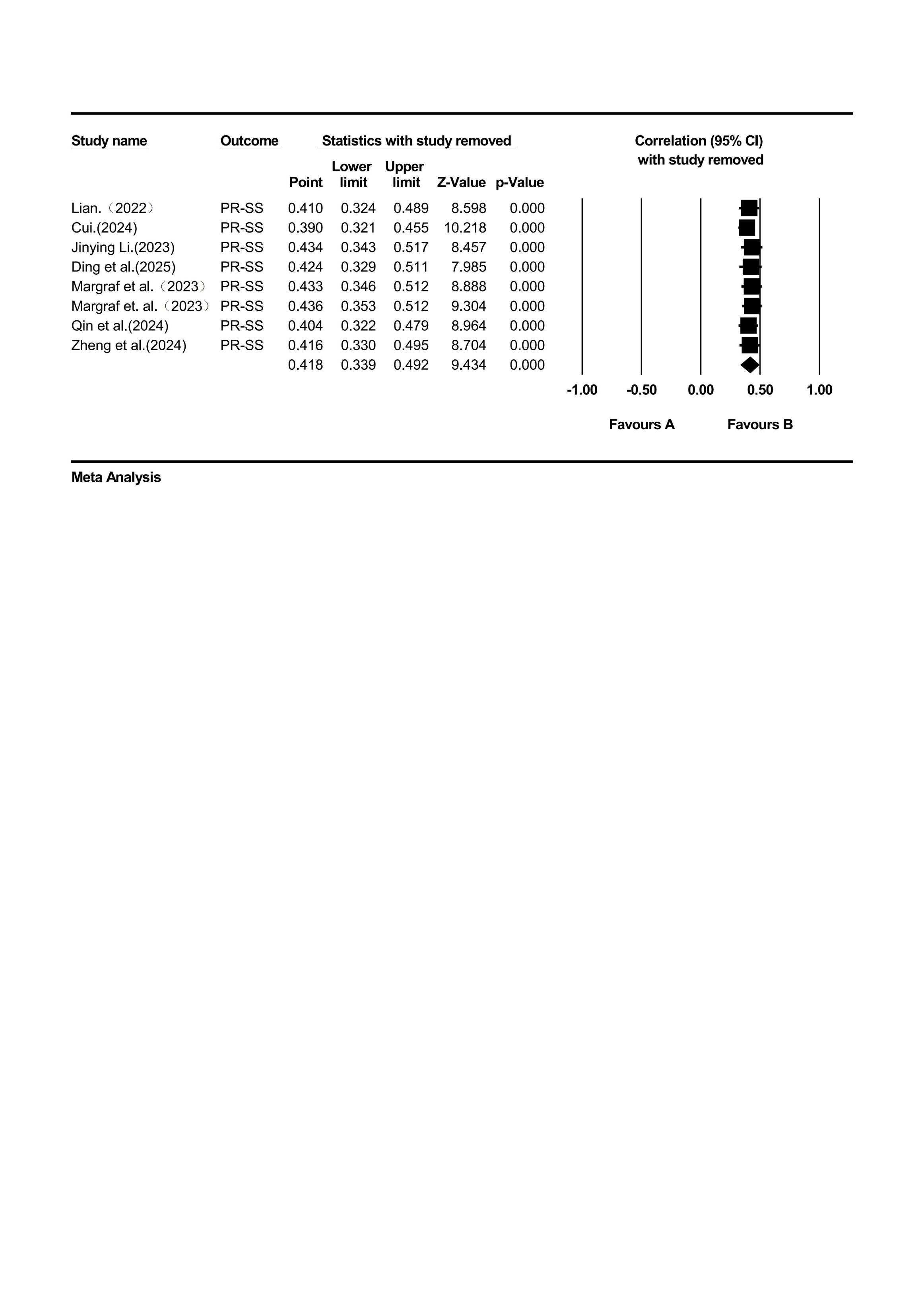
**

**Supplementary Figure 11.**Sensitivity analysis of the correlation between psychological resilience and social support
